# Biological Activity of Amidino-Substituted Imidazo [4,5-*b*]pyridines

**DOI:** 10.3390/molecules28010034

**Published:** 2022-12-21

**Authors:** Ida Boček Pavlinac, Katarina Zlatić, Leentje Persoons, Dirk Daelemans, Mihajlo Banjanac, Vedrana Radovanović, Kristina Butković, Marijeta Kralj, Marijana Hranjec

**Affiliations:** 1 Faculty of Chemical Engineering and Technology, University of Zagreb, 10000 Zagreb, Croatia; 2 Division of Molecular Medicine, Institute Ruđer Bošković, 10000 Zagreb, Croatia; 3 KU Leuven Department of Microbiology, Immunology and Transplantation, Laboratory of Virology and Chemotherapy, Rega Institute, 3000 Leuven, Belgium; 4 Pharmacology In Vitro, Selvita Ltd., 10000 Zagreb, Croatia; 5 Chemistry, Selvita Ltd., 10000 Zagreb, Croatia

**Keywords:** amidines, antibacterial activity, antiproliferative activity, antiviral activity, imidazo[4,5-*b*]pyridines

## Abstract

A series of cyano- and amidino-substituted imidazo[4,5-*b*]pyridines were synthesized using standard methods of organic synthesis, and their biological activity was evaluated. Biological evaluation included in vitro assessment of antiproliferative effects on a diverse selection of human cancer cell lines, antibacterial activity against chosen Gram-positive and Gram-negative bacterial strains, and antiviral activity on a broad panel of DNA and RNA viruses. The most pronounced antiproliferative activity was observed for compound **10**, which contained an unsubstituted amidino group, and compound **14**, which contained a 2-imidazolinyl amidino group; both displayed selective and strong activity in sub-micromolar inhibitory concentration range against colon carcinoma (IC_50_ 0.4 and 0.7 μM, respectively). All tested compounds lacked antibacterial activity, with the exception of compound **14**, which showed moderate activity against *E. coli* (MIC 32 μM). Bromo-substituted derivative **7**, which contained an unsubstituted phenyl ring (EC_50_ 21 μM), and *para*-cyano-substituted derivative **17** (EC_50_ 58 μM) showed selective but moderate activity against respiratory syncytial virus (RSV).

## 1. Introduction

Heterocycles are important building blocks in the rational design of novel biologically active molecules in medicinal and pharmaceutical chemistry due to their structural and chemical diversity and the fact that they are utilized throughout many biochemical processes [1,2]. One of the great benefits of heterocyclic chemistry is the fact that there are numerous ways to manipulate such structures in order to change the type and number of heteroatoms, the size of the ring or the incorporation of functional groups as substituents or as part of the ring itself [3,4]. Thus, the structure of heterocyclic derivatives can be optimized towards more biologically active, selective and stable molecules with potential medicinal applications [5,6].

**Figure 1:**
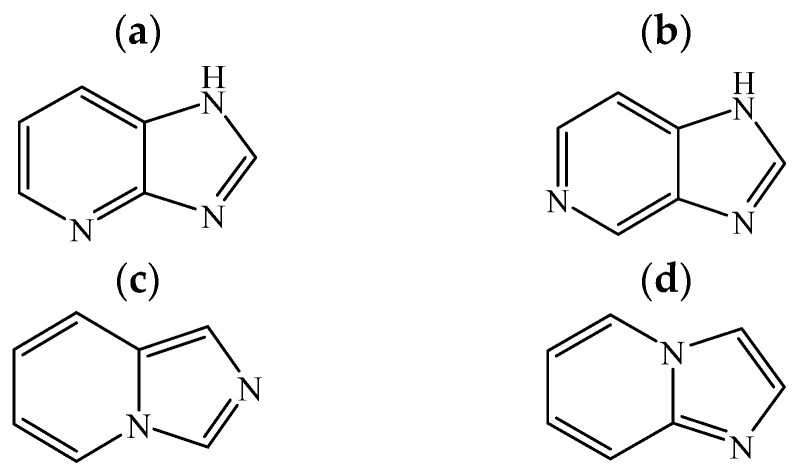
Isomer forms of imidazopyridines. (**a**) imidazo[4,5-*b*]pyridine; (**b**) imidazo[4,5-*c*]pyridine; (**c**) imidazo[1,5-*a*]pyridine; (**d**) imidazo[1,2-*a*]pyridine.

Among all known heterocycles, nitrogen-containing heterocycles are one of the most important subclasses, with benzimidazole, benzothiazole, benzoxazole and imidazopyridine being the most important ones [7,8]. Consequently, nitrogen heterocycles have been the subject of intensive studies in organic and medicinal chemistry [8,9]. Due to their presence in many natural and synthetic molecules and the fact that they possess a wide range of different biological features, nitrogen heterocycles have been selected as building blocks for the synthesis of various therapeutic small molecules in drug design [10,11]. Imidazopyridines are nitrogen heterocycles containing a pyridine and an imidazole ring in their structure. They can exist as several isomer forms as presented in Figure 1, with imidazo[4,5-*b*]pyridine, imidazo[4,5-*c*]pyridine and imidazo[1,2-*a*]pyridine being the most studied derivatives [12].

Imidazopyridine derivatives have great therapeutic potential, and they play an important role in the treatment of numerous diseases due to the wide range of their biological activities [13,14,15]. Because the structure of the abovementioned isomers is similar to naturally occurring purines, imidazopyridine derivatives can also easily interact with essential biomolecules of living systems.

**Figure 2:**
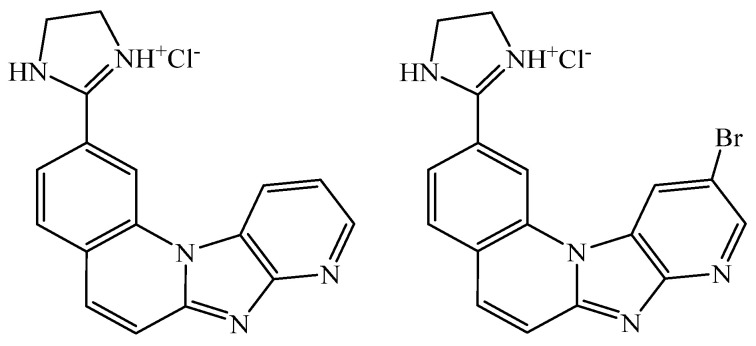
Tetracyclic imidazo[4,5-*b*]pyridine derivatives substituted with a cyclic amidino group exert potent antiproliferative activity.

Among the most important biological properties displayed by imidazopyridine derivatives are antitumor [16,17,18], antibacterial [19,20], antiviral [21] and anti-inflammatory [22] activity. Recently, we have published several studies involving the synthesis, biological evaluation and spectroscopic properties [23] of various imidazo[4,5-*b*]pyridine derivatives. We confirmed the antioxidative potential of novel unsubstituted and *N*-substituted imidazo[4,5-*b*]pyridine derived acrylonitriles, with some derivatives displaying significantly improved activity compared with reference standard BHT [24]. Additionally, imidazo[4,5-*b*]pyridine-derived acrylonitriles were explored for their in vitro antiproliferative effects on a diverse panel of human cancer cell lines, which yielded three compounds with potent sub-micromolar activity (IC_50_ 0.2–0.6 μM). Immunofluorescence staining and in vitro tubulin polymerization assays confirmed that tubulin was the main target. The most active lead compound potently inhibited cancer cell proliferation and migration while not affecting the viability of normal cells even at the highest concentration tested, making it a very promising anticancer agent [25]. Additionally, we have synthesized substituted imidazo[4,5-*b*] derivatives and their tetracyclic triaza-benzo[*c*]fluorenes, which demonstrated prominent antitumoral effects, with the amidino-substituted derivatives being the most active (Figure 2). 

The strongest broad-spectrum antiproliferative effect was observed for triaza-benzo[*c*]fluorenes bearing a 2-imidazolinyl moiety, which exert their activity by intercalating into dsDNA [26]. Following up on our previously published work on the biological activity of imidazo[4,5-*b*]pyridines and the fact that the amidino-substituted imidazo[4,5-*b*]pyridines are underexplored but promising pharmaceuticals, we have now designed and prepared a novel series of imidazo[4,5-*b*]pyridine derivatives bearing amidino substituents to explore their antiproliferative, antibacterial and antiviral activity.

## 2. Results and Discussion

### 2.1. Chemistry

**Scheme 1:**
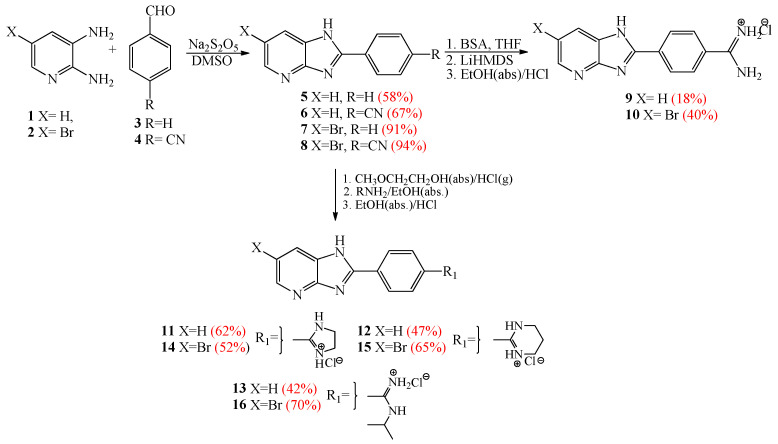
Synthesis of amidino-substituted imidazo[4,5-*b*]pyridines **9**–**10** and **11**–**16**.

Novel amidino-substituted 2-phenyl-substituted imidazo[4,5-*b*]pyridines **9**–**10** and **11**–**16** were synthesized as presented in Scheme 1.

**Scheme 2:**
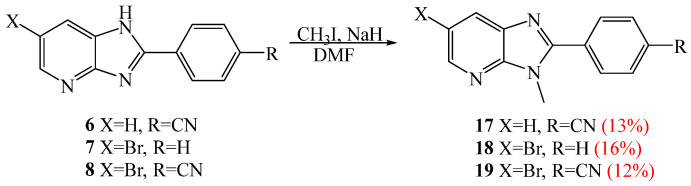
Synthesis of *N*-methyl-substituted imidazo[4,5-*b*]pyridines **17**–**19**.

Precursors **5**–**8** were prepared from corresponding 2,3-diaminopyridines **1**–**2** and benzaldehydes **3**–**4** in moderate to high yields (58–94%) using DMSO-mediated cyclization in the presence of Na_2_S_2_O_5_. Targeted compounds **9** and **10** bearing an unsubstituted amidino group were synthesized by a one-pot procedure. The previously described method [27] included protection of the NH proton using *N,O*-bis(trimethylsilyl)acetamide (BSA), nucleophilic addition of lithium hexamethyldisilazane (LiHMDS) and HCl work-up that resulted in deprotected amidines isolated as hydrochloric salts in low to moderate yields (18 and 40%). The reaction was monitored by the appearance of two singlets related to the amino protons in the 9.61–9.37 ppm range. Substituted amidino compounds **11**–**16** were prepared using a two-step Pinner reaction in 2-methoxyethanol. The progress of the first reaction step was monitored by IR spectroscopy in order to determine the conversion of cyano-substituted analogues **6** and **8** to imino ester. The targeted amidines were synthesized in the second reaction step from imino ester and an excess of corresponding amines, which gave moderate yields (42–70%). The appearance of peaks in the 10.91-10.31 ppm range and in the aliphatic region of the ^1^H NMR spectrum related to the protons of the amino group confirmed the formation of the substituted amidino compounds. All amidino-substituted compounds were prepared as hydrochloride salts in order to achieve better solubility. All structures were confirmed by ^1^H and ^13^C NMR spectroscopy, mass spectrometry and elemental analysis. *N*-methyl-substituted derivatives **17**–**19** presented in Scheme 2 were prepared in low yields (12–16%), so the desired Pinner reaction to prepare the corresponding amidino-substituted analogues was not conducted. The alkylation reaction on the imidazo[4,5-*b*]pyridine core is not selective and often results in different monoalkylated and polyalkylated products [28,29]. Successful alkylation using methyl-iodide in DMF was confirmed by the appearance of signals in the 3.97–3.95 ppm range related to the protons of the methyl group.

### 2.2. Biological Activity

In order to explore the biological potential of amidino-substituted compounds **9**–**10** and **11**–**16**, and *N*-methyl-substituted **17**–**19** imidazo[4,5-*b*]pyridine derivatives, their *in vitro* antiproliferative, antibacterial and antiviral activity was evaluated.

#### 2.2.1. Antiproliferative Activity In Vitro

Precursors **5**–**8** (except for derivative **7** due to low solubility) and all synthesized imidazo[4,5-*b*]pyridines **9**–**10**, **11**–**16** and **17**–**19** were tested for their in vitro antiproliferative activity on a diverse selection of human cancer cell lines: LN-229—glioblastoma, Capan-1—pancreatic adenocarcinoma, HCT-116—colorectal carcinoma, NCI-H460—lung carcinoma, DND-41—acute lymphoblastic leukemia, HL-60—acute myeloid leukemia, K-562—chronic myeloid leukemia and Z-138—non-Hodgkin lymphoma cancer cells (Table 1). 

Additionally, all synthesized compounds were tested against SW620—colon carcinoma, PC3—prostate carcinoma and HeLa—cervical carcinoma human cancer cells (Table 2).

The results are displayed in Table 1 and Table 2 as IC_50_ values (50% inhibitory concentrations) and are compared to docetaxel (DTX), staurosporine (STS), doxorubicine and etoposide, which are well-characterized antiproliferative agents. As presented in Table 1, the series of derivatives showed moderate activity against this panel of cancer cell lines, with the bromo-substituted compound **14** bearing an amidino 2-imidazolinyl group having the most potent effect, with IC_50_ values ranging from 8.0 to 49.7 μM. The most pronounced effect for compound **14** was observed against glioblastoma (IC_50_ 8.0 μM), non-Hodgkin lymphoma (IC_50_ 8.4 μM), pancreatic adenocarcinoma (IC_50_ 9.4 μM) and acute myeloid leukemia cells (IC_50_ 9.5 μM). Compound **15** substituted with a hexacyclic amidino group showed selective activity against acute lymphoblastic leukemia (IC_50_ 17.0 μM). Among the amidino- and bromo-substituted imidazo[4,5-*b*]pyridines, compound **10** bearing an unsubstituted amidino group did not show any activity at all, while isopropyl-amidino-substituted derivative **16** showed activity against acute lymphoblastic leukemia (IC_50_ 11.9 μM) and non-Hodgkin lymphoma (IC_50_ 12.1 µM). All tested compounds proved less potent than the reference compounds DTX and STS. In summary, these results demonstrated that the strongest impact on the proliferation of these cancer cell lines was exerted by the pentacyclic, 2-imidazolinyl amidino group, with compound **14** being the most effective when compared with the other two amidino-substituted derivatives **15**–**16**. Of these, compound **16** substituted with an isopropyl amidino group displayed a broader activity range in comparison with compound **15** bearing a hexacyclic amidino group. Additionally, the unsubstituted bromo derivatives **11**–**13** proved significantly less active in comparison with the bromo-substituted derivatives **14**–**16**.

Additionally, since some of our previously synthesized benzazole derivatives substituted with different types of amidino substituents showed promising and selective activity against colon adenocarcinoma SW620 and endocervical adenocarcinoma HeLa cells [23,30,31], and since some pentamidine analogues were reported to strongly inhibit the proliferation of prostate cancer [32], all synthesized compounds were tested against SW620, HeLa and PC3 cancer cell lines (Table 2).

**Figure 3:**
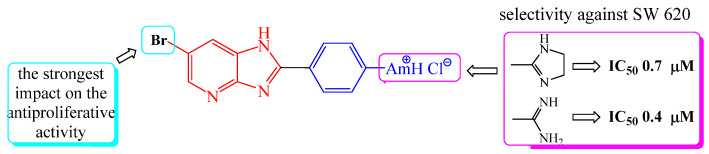
Structure–activity relationship regarding in vitro antiproliferative activity.

The results were compared to etoposide and doxorubicine as standard antitumor drugs. All derivatives showed moderate to strong antiproliferative activity against at least one of these cell lines, with the exception of compounds **9**, **12**, **13** and **17**. Compounds **5**, **6** and **18** displayed selectivity towards HeLa cells, while compounds **10** and **11** showed activity against both HeLa and SW620 cells. The bromo-substituted imidazo[4,5-*b*]pyridine **8** bearing a 4-cyanophenyl group at position 2 potently inhibited proliferation of all three cell lines (IC_50_ 1.8–3.2 μM). Generally, we can conclude that the substitution of the pyridine nuclei with bromine markedly increased the antiproliferative activity of the tested imidazo[4,5-*b*]pyridines (Figure 3). The most promising activity was observed for compound **10**, a bromo-substituted derivative bearing an unsubstituted amidino group at the phenyl ring, and (once again) compound **14**, the bromo-substituted derivative bearing a 2-imidazolinyl group at the phenyl ring. Both derivatives showed inhibitory activity at sub-micromolar concentrations against colon carcinoma (SW620), with IC_50_ values of 0.4 and 0.7 µM, respectively. The *N*-methyl-substituted derivative **18** showed decreased antiproliferative activity in comparison with the *N*-unsubstituted analogues **6**–**8**.

#### 2.2.2. Antibacterial Activity In Vitro

The bactericidal activity of all synthesized derivatives was evaluated against four different bacterial strains (Table 3).

The Gram-positive bacterial strains comprised *S. aureus* and *S. pneumoniae*, while the panel of Gram-negative bacteria included two different strains of *E. coli*. As reference drugs, four antibiotics (*ampicillin*, *ceftazidime, ciprofloxacin* and *meropenem*) were included. Based on the obtained results, we can conclude that all tested imidazo[4,5-*b*]pyridine derivatives were devoid of antibacterial activity, with MIC values > 64 μM (Table 3). Derivative **14** substituted with bromine at the pyridine nuclei and bearing a 2-imidazolinyl group at the phenyl ring showed moderate activity against *E. coli* (MIC 32 μM).

#### 2.2.3. Cytotoxicity and Antiviral Activity In Vitro

All prepared imidazo[4,5-*b*]pyridines **5**–**19** were also tested for their cytotoxicity on three virus host cell lines (HEL 299, Huh7 and MDCK) as well as their antiviral activity against several viruses (human coronavirus 229E, NL63 and OC43; influenzavirus A and B; respiratory syncytial virus; herpes simplex virus-1; yellow fever virus; Zika virus and sindbis virus), as depicted in Table 4 and Table 5. The results are expressed as CC_50_ (50% cytotoxic concentration) and EC_50_ (50% effective concentration) values, respectively, and all inactive derivatives were excluded from Table 5. For comparison of the results, five standard antiviral drugs were included. To assess the possible antiviral activity of the synthesized derivatives, their cytotoxic effect on the virus host cell lines was first determined using the colorimetric MTS assay. No negative impact on the cell viability was measured for the majority of the tested compounds, as can be seen in Table 4. Derivatives **5**, **11**, **13** and **15** were mildly cytotoxic, with CC_50_ values above 30 µM on either one or two of the tested host cell lines.

The most pronounced cytotoxicity was seen for the bromo-substituted compound **14** bearing a 2-imidazolinyl amidino group, which had a relatively high CC_50_ on the MDCK cell line (33.9 μM) but CC_50_ values of 9.3 µM and even below 0.8 µM on HEL 299 and Huh7 cells, respectively. Similar to the antiproliferation results, among amidino- and bromo-substituted imidazo[4,5-*b*]pyridines, compound **10** bearing an unsubstituted amidino group did not show any cytotoxicity at all, while isopropyl-amidino-substituted derivative **16** showed mild toxicity on MDCK (CC_50_ 71.2 µM) and a more pronounced cytotoxic effect on Huh7 cells (CC_50_ 3.0 µM). The whole set of newly synthesized compounds was tested in vitro against a panel of DNA and RNA viruses. Reference antiviral drugs remdesivir, ribavirin, zanamivir, rimantadine and brivudine (BVDU) were included in the CPE reduction assays as positive controls. Overall, no significant broad-spectrum antiviral activity was detected for the imidazo[4,5-*b*]pyridine derivatives. Compound **8**, a bromo-substituted imidazo[4,5-*b*]pyridine substituted with a 4-cyanophenyl group at position two, as well as compound **10**, a bromo-substituted derivative bearing an unsubstituted amidino group at the phenyl ring, both showed weak but broad anti-influenza virus activity, inhibiting all tested subtypes (H1N1, H3N2 and B). The most promising antiviral activity was seen for bromo-substituted derivative **7** with an unsubstituted phenyl ring showing selective activity against RSV (EC_50_ 21.0 μM) as well as for *para*-cyano-substituted derivative **17** (EC_50_ 79.0 μM).

## 3. Conclusions

In this study, we describe the synthesis, structural characterization and biological evaluation of imidazo[4,5-*b*]pyridine derivatives substituted either with bromo, cyano or acyclic and cyclic amidino groups at the phenyl ring. Unsubstituted derivatives were prepared to study the influence of the type of substituent on the biological activity. We used previously published and well-optimized procedures for the synthesis of amidino derivatives from cyano precursors via a Pinner reaction or through reaction with lithium hexamethyldisilazane (LiHMDS).

All synthesized imidazo[4,5-*b*]pyridines were evaluated for their in vitro biological activity including antiproliferative activity, antibacterial activity and antiviral activity.

Antiproliferative activity was evaluated on a diverse panel of human cancer cell lines and on HeLa, PC3 and SW620 cell lines, which were included because earlier publications pointed out that amidino-substituted derivatives could be active against these three cell lines. Overall, the in vitro evaluation of antiproliferative activity revealed that the most significant impact on cancer cell proliferation was detected for the compound in which bromine was placed at the pyridine nuclei as well as for the cyclic amidino group, namely the 2-imidazolinyl group placed at the *para* position on the phenyl ring. Furthermore, compounds **10** and **14** substituted with either an unsubstituted amidino or a 2-imidazolinyl group, proved to be the most promising derivatives for pronounced and selective activity against SW620 cells (IC_50_ 0.4 and 0.7 μM, respectively). These two compounds have been chosen as lead compounds for further optimization of the presented skeleton. The prepared imidazo[4,5-*b*]pyridines **5**–**19** showed little or no antibacterial activity against four chosen bacterial strains. Additionally, all compounds were evaluated for their antiviral activity against several viruses. Four compounds showed antiviral activity, with derivatives **7** and **17** showing selective antiviral activity against respiratory syncytial virus (RSV).

In conclusion, we have shown that imidazo[4,5-*b*]pyridines bearing either a bromine at the pyridine nuclei or an amidine group at the phenyl ring have promising biological potential with pronounced antiproliferative activity. All presented results point out that this chemical class harbors promising candidates for further design and optimization in order to developed potent antiproliferative agents.

## 4. Materials and Methods

### 4.1. General Methods

Melting points were determined using an Original Kofler Mikroheitztisch apparatus (Reichert, Wien, Austria). The ^1^H NMR and the ^13^C NMR spectra were recorded with the Bruker Avance DPX-300 or Bruker AV-600 using TMS as an internal standard. Chemical shifts were reported in parts per million (ppm) relative to TMS. Elemental analyses for carbon, hydrogen and nitrogen were performed on PerkinElmer 2400 elemental analyzer. Analyses were indicated as symbols of elements, and analytical results obtained were within 0.4% of the theoretical value. All compounds were routinely checked by TLC using Merck silica gel 60F-254 glass plates. All NMR spectra are given in Supplementary Materilas. 

### 4.2. Synthesis

#### 4.2.1. General Procedure for Preparation of Compounds **5**–**8**

Compounds **5**–**8** were prepared by heating equimolar amounts of 2,3-pyridines **1**–**2** and benzaldehyde derivatives **3**–**4** in the presence 0.55 equivalents of Na_2_S_2_O_5_ in DMSO at 165 °C in an oil bath.

After 15 min, the reaction mixture was cooled to room temperature and excess water was added. After 10 min, the resulting precipitate was separated by filtration.

##### 2-Phenyl-1H-imidazo[4:5-b]pyridine **5** 

Compound **5** was synthesized following the general method from compound **1** (0.44 g, 4.00 mmol), **3** (0.43 g, 4.00 mmol) and Na_2_S_2_O_5_ (0.42 g, 2.20 mmol) in DMSO (4 mL) to obtain 0.44 g (55.7%) of light brown powder; m.p. 290–291 °C. ^1^H NMR (DMSO-*d*_6_, 600 MHz): δ/ppm = 13.47 (s, 1H, NH), 8.36 (dd, 1H, *J_AB_* = 4.71 Hz, *J_AC_* = 0.93 Hz, H_pyridine_), 8.25 (d, 2H, *J_DE_* = 7.08 Hz, H_arom_), 8.03 (d, 1H, *J_CB_* = 7.68 Hz, H_pyridine_), 7.60–7.55 (m, 3H, H_arom_), 7.26 (dd, 1H, *J_BC_* = 7.92 Hz, *J_BA_* = 4.74 Hz, H_pyridine_); ^13^C NMR (DMSO-*d*_6_, 151 MHz): δ/ppm = 153.21, 144.23, 131.03, 130.08 (2C), 129.50 (2C), 127.21 (2C), 118.57. MS (ESI): *m*/*z* = 196.64 ([M+H]^+^). Anal. Calcd. for C_12_H_9_N_3_: C, 73.83; H, 4.65; N, 21.52. Found: C, 73.38; H, 4.69; N, 21.49%.

##### 4-(1H-Imidazo[4,5-b]pyridin-2-yl)benzonitrile **6**

Compound **6** was synthesized following the general method from **1** (0.22 g, 2.00 mmol), **4** (0.26 g, 2.00 mmol) and Na_2_S_2_O_5_ (0.21 g, 1.10 mmol) in DMSO (2 mL) to obtain 0.29 g (67.1%) of beige powder; m.p. > 300 °C. ^1^H NMR (DMSO-*d*_6_, 600 MHz): δ/ppm = 13.82 (bs, 1H, NH), 8.41 (d, 3H, *J_DE_* = 8.46 Hz, H_arom,pyridine_), 8.11 (s, 1H, H_pyridine_), 8.04 (d, 2H, *J_ED_* = 8.34 Hz, H_arom_), 7.31 (dd, 1H, *J_BA_* = 8.04 Hz, *J_BC_* = 4.74 Hz, H_pyridine_); ^13^C NMR (DMSO-*d*_6_, 151 MHz): δ/ppm = 145.21, 134.13, 133.39 (2C), 127.72, 119.10, 118.88 (2C), 113.11. MS (ESI): *m*/*z* = 221.06 ([M+H]^+^). Anal. Calcd. for C_13_H_8_N_4_: C, 70.90; H, 3.66; N, 25.44. Found: C, 70.85; H, 3.70; N, 25.47%.

##### 6-Bromo-2-phenyl-1H-imidazo[4,5-b]pyridine **7**

Compound **7** was synthesized following the general method from **2** (1.00 g, 5.31 mmol), **3** (0.56 g, 5.31 mmol) and Na_2_S_2_O_5_ (0.55 g, 2.92 mmol) in DMSO (5 mL) to obtain 1.33 g (91.4%) of light brown powder; m.p. > 300 °C. ^1^H NMR (DMSO-*d*_6_, 400 MHz): δ/ppm = 8.43 (d, 1H, *J_AC_* = 2.12 Hz, H_pyridine_), 8.28 (d, 1H, *J_CA_* = 1.52 Hz, H_pyridine_), 8.26–8.22 (m, 2H, H_arom_), 7.59–7.57 (m, 3H, H_arom_); ^13^C NMR (DMSO-*d*_6_, 151 MHz): δ/ppm = 154.67, 144.57, 131.43, 129.62, 129.56 (2C), 127.40 (2C), 113.46. MS (ESI): *m*/*z* = 275.99/277.13 ([M+H]^+^). Anal. Calcd. for C_12_H_8_BrN_3_: C, 52.58; H, 2.94; N, 15.33. Found: C, 52.61; H, 2.94; N, 15.35%.

##### 4-(6-Bromo-1H-imidazo[4,5-b]pyridin-2-yl)benzonitrile **8**

Compound **8** was synthesized following the general method from **2** (0.75 g, 4.00 mmol), **4** (0.26 g, 2.00 mmol) and Na_2_S_2_O_5_ (0.21 g, 2.20 mmol) in DMSO (2 mL) to obtain 1.12 g (93.8%) of beige powder; m.p. > 300 °C. ^1^H NMR (DMSO-*d*_6_, 400 MHz): δ/ppm = 14.04 (bs, 1H, NH), 8.48 (d, 1H, *J_AC_* = 2.08 Hz, H_pyridine_), 8.38 (d, 2H, *J_DE_* = 8.56 Hz, H_arom_), 8.37 (d, *J_CA_* = 2.20 Hz, H_pyridine_) 8.06 (d, 2H, *J_ED_* = 8.52 Hz, H_arom_); ^13^C NMR (DMSO-*d*_6_, 101 MHz): δ/ppm = 152.76, 145.52, 133.80, 133.55 (2C), 127.96 (2C), 118.92, 114.00, 113.40. MS (ESI): *m*/*z* = 298.97/300.05 ([M+H]^+^). Anal. Calcd. for C_13_H_7_BrN_4_: C, 52.20; H, 2.36; N, 18.73. Found: C, 52.12; H, 2.37; N, 18.68%.

#### 4.2.2. General Method for Preparation of Compounds **9**–**10**

Equivalents of BSA were added to the suspension of **6** or **8** in anhydrous THF 1.1, and the reaction mixture was stirred under argon for 30 min. The reaction mixture was cooled to 0 °C, and 15 equivalents of LiHMDS solution (1M in THF) were added dropwise. After completion of addition, the reaction mixture was stirred for 6 h at room temperature, cooled to 0 °C and quenched with 4 M HCl in ethanol. The solvent was removed under pressure, and solid residue was treated with ethanol and 4 M HCl in ethanol until the mixture became acidic. Additional ethanol–HCl was added, and the mixture was stirred for 2 h. After removing the solvent under reduced pressure, diethyl ether was added, and the solid was separated by filtration. The solid was suspended in water and treated with 20% NaOH to pH 9. The mixture was filtered, and the air-dried solid was suspended in ethanol and treated with HCl. Diethyl ether was added and the solid was separated by filtration, which gave amidine HCl salt.

##### 4-(1H-Imidazo[4,5-b]pyridin-2-yl)benzimidamide Hydrochloride **9**

Compound **9** was synthesized following the general method from **6** (0.11 g, 0.50 mmol), BSA (134 µL, 0.55 mmol) in 1 mL of THF and LiHDMS (7.5 mL, 7.50 mmol) to obtain 0.03 g (17.5%) of white powder; m.p. > 300 °C. ^1^H NMR (DMSO-*d*_6_, 400 MHz): δ/ppm = 9.61 (s, 2H, NH_amidine_), 9.39 (s, 2H, NH_amidine_), 8.60 (dd, 1H, *J_AB_* = 5.36 Hz, *J_AC_* = 1.04 Hz, H_pyridine_), 8.57 (d, 2H, *J_DE_* = 8.48 Hz, H_arom_), 8.43 (d, 1H, *J_CB_* = 7.88 Hz, H_pyridine_), 8.09 (d, 2H, *J_ED_* = 8.48 Hz, H_arom_), 7.58 (dd, 1H, *J_BC_* = 7.90 Hz, *J_BA_* = 5.38 Hz, H_pyridine_); ^13^C NMR (DMSO-*d*_6_, 151 MHz): δ/ppm = 165.47, 149.88, 133.18, 130.96, 129.61 (2C), 128.19 (2C), 127.12, 119.61. MS (ESI): *m*/*z* = 236.24 ([M+H]^+^) Free base: 236.24. Anal. Calcd. for C_13_H_12_ClN_5_: C, 57.04; H, 4.42; N, 25.59. Found: C, 56.98; H, 4.41; N, 25.63%.

##### 4-(6-Bromo-1H-imidazo[4,5-b]pyridin-2-yl)benzimidamide Hydrochloride **10**

Compound **10** was synthesized following the general method from **8** (0.15 g, 0.50 mmol), BSA (134 µL, 0.55 mmol) in 1 mL of THF and LiHDMS (7.5 mL, 7.50 mmol) to obtain 0.07 g (39.8%) of light brown powder; m.p. > 300 °C; ^1^H NMR (DMSO-*d*_6_, 400 MHz): δ/ppm = 9.58 (s, 2H, NH_amidine_), 9.37 (s, 2H, NH_amidine_), 8.58 (d, 1H, *J_AC_* = 2.12 Hz, H_pyridine_), 8.48 (d, 2H, *J_DE_* = 8.60 Hz, H_arom_), 8.35 (d, 1H, *J_CA_* = 2.16 Hz, H_pyridine_), 8.05 (d, 2H, *J_ED_* = 8.64 Hz, H_arom_); ^13^C NMR (DMSO-*d*_6_, 151 MHz): δ/ppm = 165.52, 152.97, 145.46, 134.05, 130.27, 129.47(2C), 127.68(2C), 114.02. MS (ESI): *m*/*z* = 315.95/317.82 ([M+H]^+^). Free base 316.16. Anal. Calcd. for C_13_H_11_BrClN_5_: C, 44.28; H, 3.14; N, 19.86. Found: C, 44.33; H, 3.15; N, 19.90%.

##### 4.2.3. General Method for Preparation of Compounds **11**–**16**

A nitrile compound was suspended in anhydrous 2-methoxyethanol and cooled to 0 °C. Dry HCl gas was bubbled into a cooled suspension for 4 h. The suspension was monitored with IR spectroscopy and stirred at room temperature until the –CN band was undetectable. Anhydrous diethyl ether was added to the suspension and the solid was filtered off and dried in vacuo. The dry product was suspended in anhydrous ethanol, and corresponding amine was added. The crude product was filtered off and washed with diethyl ether to give powder products, which were suspended in anhydrous ethanol and concentrated HCl was added. For the synthesis of imidazolinyl-substituted amidines **11** and **14**, 3.5 equivalents of ethylendiamine were added to a suspension of imidate ester, and the reaction was heated for 24 h. For the synthesis of 1,4,5,6-tetrahydropyrimidine-substituted amidines **12** and **15**, two equivalents of 1,3-propylenediamine were added to a suspension of imidate ester, and the reaction was heated for 24 h. For the synthesis of isopropyl-substituted amidines **13** and **16**, five equivalents of isopropyl amine were added to a suspension of imidate ester, and the reaction was heated for 24 h.

###### 2-(4-(4,5-Dihydro-1H-imidazol-2-yl)phenyl)-1H-imidazo[4,5-b]pyridine Hydrochloride **11**

Compound **11** was synthesized from **6** (0.50 g, 2.27 mmol) and ethylendiamine (210 µL, 3.16 mmol) to yield 0.04 g (62.2%) of white powder; m.p. > 300 °C. ^1^H NMR (DMSO-*d*_6_, 600 MHz): δ/ppm = 13.90 (bs, 1H, NH), 10.91 (s, 2H, NH_amidine_), 8.50 (s, 2H, H_arom_), 8.43 (s, 1H, H_pyridine_), 8.23 (d, 2H, *J_ED_* = 8.40 Hz, H_arom_), 8.08 (s, 1H, H_pyridine_), 7.31 (dd, 1H, *J_BC_* = 7.80 Hz, *J_BA_* = 4.74 Hz, H_pyridine_), 4.04 (s, 4H, CH_2_); ^13^C NMR (DMSO-*d*_6_, 151 MHz): δ/ppm = 164.70, 145.28, 135.32, 129.82 (2C), 127.60, 123.87, 119.15, 44.93 (2C). MS (ESI): *m*/*z* = 263.93 ([M+H]^+^). Free base: 263.30. Anal. Calcd. for C_15_H_14_ClN_5_: C, 60.10; H, 4.71; N, 23.36. Found: C, 60.21; H, 4.68; N, 23.30%.

###### 2-(4-(1H-Imidazo[4,5-b]pyridin-2-yl)phenyl)-3,4,5,6-tetrahydropyrimidin-1-ium Chloride **12**

Compound **12** was synthesized from **6** (0.50 g, 2.27 mmol) and 1,3-propylenediamine (151 µL, 1.80 mmol) to yield 0.029 g (52%) of white powder; m.p. > 300 °C. ^1^H NMR (DMSO-*d*_6_, 600 MHz): δ/ppm = 10.38 (s, 2H, NH_amidine_), 8.60 (dd, 1H, *J_AB_* = 5.28 Hz, *J_AC_* = 1.08 Hz, H_pyridine_), 8.58 (d, 2H, *J_DE_* = 8.52 Hz, H_arom_), 8.46 (d, 1H, *J_CB_* = 7.92 Hz, H_pyridine_), 8.04 (d, 2H, *J_ED_* = 8.58 Hz, H_arom_), 7.60 (dd, 1H, *J_BC_* = 7.88 Hz, *J_BA_* = 5.46 Hz, H_pyridine_), 3.53 (m, 4H, CH_2_), 2.01 (m, 2H, CH_2_); ^13^C NMR (DMSO-*d*_6_, 151 MHz): δ/ppm = 158.85, 149.65, 132.51, 131.35, 129.22 (2C), 128.34 (2C), 127.25, 119.63, 18.06. MS (ESI): *m*/*z* = 278.14 ([M+H]^+^). Free base: 277.32. Anal. Calcd. for C_16_H_16_ClN_5_: C, 61.24; H, 5.14; N, 22.32. Found: C, 61.14; H, 5.11; N, 22.28%.

###### 4-(1H-Imidazo[4,5-b]pyridin-2-yl)-N-isopropylbenzimidamide Hydrochloride **13**

Compound **13** was synthesized from **6** (0.2 g, 1.02 mmol) and isopropyl amine (240 µL, 2.93 mmol) to yield 0.06 g (46,7%) of beige powder; m.p. 293-295 °C. ^1^H NMR (DMSO-*d*_6_, 600 MHz): δ/ppm = 9.81 (d, 1H, *J_FG_* = 7.92 Hz, NH_amidine_), 9.65 (s, 1H, NH_amidine_), 9.33 (s, 1H, NH_amidine_), 8.59 (d, 1H, *J_AB_* = 5.16 Hz, H_pyridine_), 8.57 (d, 2H, *J_DE_* = 8.40 Hz, H_arom_), 8.42 (d, 1H, *J_CB_* = 7.68 Hz, H_pyridine_), 7.99 (d, 2H, *J_ED_* = 8.46 Hz, H_arom_), 7.58 (dd, 1H, *J_BC_* = 7.65 Hz, *J_BA_* = 5.25 Hz, H_pyridine_), 4.14 (m, 1H, CH), 1.31 (d, 6H, *J_GF_* = 6.42 Hz, CH_3_); ^13^C NMR (DMSO-*d*_6_, 151 MHz): δ/ppm = 161.62, 149.94, 132.66, 132.11, 129.85, 128.06, 126.98, 119.57, 45.70, 31.17, 21.68 (2C). MS (ESI): *m*/*z* = 280.19 ([M+H]^+^). Free base: 279.34. Anal. Calcd. for C_16_H_18_ClN_5_: C, 60.85; H, 5.75; N, 22.18. Found: C, 60.90; H, 5.73; N, 22.20%.

###### 6-Bromo-2-(4-(4,5-dihydro-1H-imidazol-2-yl)phenyl)-1H-imidazo[4,5-b]pyridine Hydrochloride **14**

Compound **14** was synthesized from **8** (0.50 g 1.67 mmol) and ethylendiamine (114 µL, 2.71 mmol) to yield 0.06 g (64,6%) of off-white powder; m.p. > 300 °C. ^1^H NMR (DMSO-*d*_6_, 600 MHz): δ/ppm = 10.85 (s, 2H, NH_amidine_), 8.50 (d, 1H, *J_AC_* = 2.16 Hz, H_pyridine_), 8.49 (d, 2H, *J_DE_* = 8.58 Hz, H_arom_), 8.37 (s, 1H, H_pyridine_), 8.21 (d, 2H, *J_ED_* = 8.64 Hz, H_arom_), 4.05 (s, 4H, CH_2_); ^13^C NMR (DMSO-*d*_6_, 151 MHz): δ/ppm = 164.76, 152.79, 145.60, 134.86, 129.80 (2C), 127.89 (2C), 124.22, 114.06, 56.48, 44.97 (2C). MS (ESI): *m*/*z* = 342.05/344.10 ([M+H]^+^). Free base: 342.19. Anal. Calcd. for C_15_H_13_BrClN_5_: C, 47.58; H, 3.46; N, 18.50. Found: C, 47.66; H, 3.43; N, 18.57%.

###### 6-Bromo-2-(4-(1,4,5,6-tetrahydropyrimidin-2-yl)phenyl)-1H-imidazo[4,5-b]pyridine Hydrochloride **15**

Compound **15** was synthesized from **8** (0.50 g 1.67 mmol) and 1,3-propylenediamine (74 µL, 0.874 mmol) to yield 0.03 g (42,2%) of white powder; m.p. > 300 °C. ^1^H NMR (DMSO-*d*_6_, 600 MHz): δ/ppm = 10.31 (s, 2H, NH_amidine_), 8.51 (d, 1H, *J_AC_* = 2.04 Hz, H_pyridine_), 8.48 (d, 2H, *J_DE_* = 8.40 Hz, H_arom_), 8.35 (d, 1H, *J_CA_* = 1.98 Hz, H_pyridine_), 7.99 (d, 2H, *J_ED_* = 8.40 Hz, H_arom_.), 3.52 (s, 4H, CH_2_), 2.07–1.93 (m, 2H, CH_2_); ^13^C NMR (DMSO-*d*_6_, 151 MHz): δ/ppm = 158.94, 153.04, 145.42, 133.48, 130.65 (2C), 129.04 (2C), 127.77, 114.02, 18.08 (2C). MS (ESI): *m*/*z* = 356.05/358.11 ([M + H]^+^). Free base 356.23. Anal. Calcd. for C_16_H_15_BrClN_5_: C, 48.94; H, 3.85; N, 17.83. Found: C, 49.01; H, 3.82; N, 17.78%.

###### 4-(6-Bromo-1H-imidazo[4,5-b]pyridin-2-yl)-N-isopropylbenzimidamide Hydrochloride **16**

Compound **16** was prepared from **8** (0.50 g, 1.67 mmol) and isopropyl amine (204 µL, 2.49 mmol) to yield 0.03 g (70.1%) of light brown powder; m.p. > 300 °C. ^1^H NMR (DMSO-*d*_6_, 600 MHz): δ/ppm = 14.11 (s, 1H, NH), 9.73 (d, 1H *J_FG_* = 7.80 Hz, NH_amidine_), 9.56 (s, 1H, NH_amidine_), 9.22 (s, 1H, NH_amidine_), 8.49 (d, 1H, *J_AC_* = 1.62 Hz, H_pyridine_), 8.46 (d, 2H, *J_DE_* = 8.34 Hz, H_arom_), 8.35 (s, 1H, H_pyridine_), 7.94 (d, 2H, *J_ED_* = 8.40 Hz, H_arom_), 4.14–4.03 (m, 1H, CH), 1.30 (d, 6H, *J_GF_* = 6.42 Hz, CH_3_); ^13^C NMR (DMSO-*d*_6_, 151 MHz): δ/ppm = 161.76, 153.10, 145.40, 133.75, 131.42, 129.68 (2C), 127.48 (2C), 113.92, 45.63, 21.67 (2C). MS (ESI): *m*/*z* = 359.99/361.36 ([M+H]^+^). Free base 358.24. Anal. Calcd. for C_16_H_17_BrClN_5_: C, 48.69; H, 4.34; N, 17.74. Found: C, 48.79; H, 4.29; N, 17.81%.

##### 4.2.4. General Procedure for Synthesis of Compounds **17**–**19**

Compounds **17**–**19** were prepared from equimolar amounts of **6**–**8** and CH_3_I in 10 mL of DMF in the presence of five equivalents of 60% NaH in mineral oil as a base. After 24 h at room temperature, the solvent was removed, and the product was purified by column chromatography on SiO_2_ using dichloromethane/methanol as the eluent.

###### 4-(3-Methyl-3H-imidazo[4,5-b]pyridin-2-yl)benzonitrile **17**

Compound **17** was prepared from **6** (0.50 g, 2.27 mmol), CH_3_I (141 µL, 2.27 mmol) and 0.45 g 60% NaH to yield 0.07 g 13.2%; m.p. 183–185 °C. ^1^H NMR (DMSO-*d*_6_, 600 MHz): δ/ppm = 8.45 (dd, 1H, *J_AB_* = 4.71 Hz, *J_AC_* = 1.41 Hz, H_pyridine_), 8.16–8.14 (m, 3H, H_arom, pyridine_), 8.08 (d, 2H, *J* = 1.80 Hz, H_arom_), 7.37 (dd, 1H, *J_BC_* = 7.98 Hz, *J_BA_* = 4.74 Hz, H_pyridine_), 3.97 (s, 3H, CH_3_); ^13^C NMR (DMSO-*d*_6_, 15 MHz): δ/ppm = 152.64, 149.15, 144.77, 135.05, 134.67, 133.16 (2C), 130.43, 127.71, 119.27, 118.92, 113.05, 30.82. MS (ESI): *m*/*z* = 235.16 ([M+H]^+^). Anal. Calcd. for C_14_H_10_N_4_: C, 71.78; H, 4.30; N, 23.92. Found: C, 71.85; H, 4.31; N, 24.00%.

###### 6-Bromo-3-methyl-2-phenyl-3H-imidazo[4,5-b]pyridine **18**

Compound **18** was prepared from **7** (0.50 g, 1.83 mmol), CH_3_I (125 µL, 2.01 mmol) and 0.41 g 60% NaH to yield 0.09 g 16.1%; m.p. 214–215 °C. ^1^H NMR (DMSO-*d*_6_, 600 MHz): δ/ppm = 8.50 (d, 1H, *J_AC_* = 2.10 Hz, H_pyridine_), 8.40 (d, 1H, *J_CA_* = 2.04 Hz, H_pyridine_), 7.99–7.92 (m, 2H, H_arom_), 7.66–7.60 (m, 3H, H_arom_), 3.93 (s, 3H, CH_3_); ^13^C NMR (DMSO-*d*_6_, 75 MHz): δ/ppm = 155.95, 148.09, 144.19, 136.32, 130.99, 129.84, 129.72 (2C), 129.36, 129.31 (2C), 113.64, 31.01.

MS (ESI): *m*/*z* = 287.98/289.98 ([M+H]^+^). Anal. Calcd. for C_13_H_10_BrN_3_: C, 54.19; H, 3.50; N, 14.58. Found: C, 54.28; H, 3.49; N, 14.62%.

###### 4-(6-Bromo-3-methyl-3H-imidazo[4,5-b]pyridin-2-yl)benzonitrile **19**

Compound **19** was prepared from **8** (0.50 g, 1.67 mmol), CH_3_I (104 µL, 1.67 mmol) and 0.33 g 60% NaH to yield 0.06 g 12.0%; m.p. 234–235 °C. ^1^H NMR (DMSO-*d*_6_, 600 MHz): δ/ppm = 8.55 (d, 1H, *J_AC_* = 2.04 Hz, H_pyridine_), 8.47 (d, 1H, *J_CA_* = 2.04 Hz, H_pyridine_), 8.15 (dd, 2H, *J_DE_* = 6.63 Hz, *J_DD′_* = 1.89 Hz, H_arom_), 8.09 (dd, 2H, *J_ED_* = 6.63 Hz, *J_EE′_* = 1.89 Hz, H_arom_), 3.95 (s, 3H, CH_3_); ^13^C NMR (DMSO-*d*_6_, 151 MHz): δ/ppm = 154.14, 148.00, 145.00, 136.21, 134.18, 133.21 (2C), 130.53 (2C), 129.90, 118.84, 113.97, 113.36, 31.06. MS (ESI): *m*/*z* = 312.90/314.98 ([M+H]^+^). Anal. Calcd. for C_14_H_9_BrN_4_: C, 53.70; H, 2.90; N, 17.89. Found: C, 53.21; H, 2.84 N, 17.97%.

### 4.3. Biological Activity 

#### 4.3.1. Antiproliferative Activity In Vitro

##### Cancer Cell Lines

Human cancer cell lines used in this manuscript, namely Capan-1, HCT-116, NCI-H460, LN-229, HL-60, K-562, Z-138, SW620, PC3 and HeLa, were acquired from the American Type Culture Collection (ATCC, Manassas, VA, USA), while the DND-41 cell line was purchased from the Deutsche Sammlung von Mikroorganismen und Zellkulturen (DSMZ Leibniz-Institut, Germany). Culture media were purchased from Gibco Life Technologies, USA, and supplemented with 10% fetal bovine serum (HyClone, GE Healthcare Life Sciences, Chicago, IL, USA), except for the media for SW620, PC3 and HeLa cells, which were purchased from Sigma.

##### Proliferation Assays

Adherent cell lines LN-229, HCT-116, NCI-H460 and Capan-1 cells were seeded at a density between 500 and 1500 cells per well in 384-well tissue culture plates (Greiner). After overnight incubation, cells were treated with seven different concentrations of the test compounds, ranging from 100 to 0.006 µM. Suspension cell lines HL-60, K-562, Z-138 and DND-41 were seeded at densities ranging from 2500 to 5500 cells per well in 384-well culture plates containing the test compounds at the same concentration points. Cells were incubated for 72 h with compounds and were then analyzed using the CellTiter 96^®^ AQueous One Solution Cell Proliferation Assay (MTS) reagent (Promega) according to the manufacturer’s instructions. Absorbance of the samples was measured at 490 nm using a SpectraMax Plus 384 (Molecular Devices), and OD values were used to calculate the 50% inhibitory concentration (IC_50_). Compounds were tested in two independent experiments. SW620, PC3 and HeLa cells were seeded in 96-well microtiter plates at 1 × 10^4^ to 3 × 10^4^ cells/mL, depending on the doubling times of the specific cell line. Test agents were then added in five 10-fold dilutions (10^−8^ to 10^−4^ M). Working dilutions were freshly prepared on the day of testing. After 72 h of incubation, the cell growth rate was evaluated using the MTT assay as described previously [33]. Absorbance of the samples was measured at 570 nm, and OD values were used to calculate the 50% inhibitory concentration (IC_50_). Each test was performed in quadruplicate in at least two individual experiments.

#### 4.3.2. Antibacterial Activity In Vitro

##### Materials

In addition to the synthesized compounds, the standard antibiotics ampicillin, ceftazidime, ciprofloxacin and meropenem from USP were tested. Selected bacterial strains were Gram-negative *Escherichia coli* (ATCC 25922) and Gram-positive *Staphylococcus aureus* (ATCC 29213) and *S. Pneumoniae* (ATCC 49619). *Saccharomyces cerevisiae* ATCC 7752 strain was tested as a eukaryotic model organism. Synthesized compounds were prepared as 10 mM DMSO solutions and tested in a final concentration range of 100–0.2 µM [34]. Standard antibiotics were prepared as 5 mg/mL DMSO solutions and tested in a final concentration range of 64–0.125 µg/mL.

##### Methods

Broth microdilution testing was performed according to CLSI (Clinical Laboratory Standards Institute) guidelines. MIC (minimal inhibitory concentration) values were defined as the last tested concentration of the compound at which there was no visible growth of bacteria. Inoculums for each microorganism were prepared using the direct colony suspension method, where broth solutions that achieved turbidity equivalent to 0.5 McFarland standard were additionally diluted 100× with Ca-adjusted MH media (Becton Dickinson). All test plates were incubated for 16–24 h at 37 °C. MIC values for reference antibiotics against quality control strains were used to confirm the validity of the screen according to the Clinical and Laboratory Standards Institute (CLSI) guidelines. Methods for dilution antimicrobial susceptibility tests for bacteria that grow aerobically were obtained from M07, 11th edition, 2018. and Clinical and Laboratory Standards Institute (CLSI) guidelines. Performance standards for antimicrobial susceptibility testing were obtained from M100, 28th edition, 2018.

#### 4.3.3. Antiviral Activity In Vitro

##### Host Cell Lines

HEL 299 (ATCC CCL-137; human lung fibroblast), Huh-7 (CLS-300156; human hepatoblastoma) and MDCK (Madin-Darby canine kidney cells; a kind gift from M. Matrosovich, Marburg, Germany) cells were maintained in Dulbecco’s Modified Eagle Medium (DMEM, Gibco Life Technologies) supplemented with 8% heat-inactivated fetal bovine serum (HyClone, GE Healthcare Life Sciences), 0.075% sodium bicarbonate (Gibco Life Technologies) and 1 mM sodium pyruvate (Gibco Life Technologies) and were maintained at 37 °C under 5% CO_2_.

##### Antiviral CPE Reduction Assays

Antiviral assays were performed with herpes simplex virus-1 (HSV-1 KOS), human coronavirus (HCoV-229E and -OC43) and respiratory syncytial virus A in HEL cell cultures, with sindbis virus, yellow fever virus, Zika virus and human coronavirus (HCoV-NL63) in Huh-7 cell cultures and with influenza A/H1N1 (A/Ned/378/05), influenza A/H3N2 (A/HK/7/87) and influenza B (B/Ned/537/05) in MDCK cell cultures. On the day of the infection, growth medium was aspirated and replaced by serial dilutions of the test compounds. The virus was then added to each well, diluted to obtain a viral input of 100 CCID_50_ (CCID_50_ being the virus dose that is able to infect 50% of the cells). Mock-treated cultures receiving solely the test compounds were included to determine the cytotoxicity. After three to seven days of incubation, the virus-induced cytopathogenic effect was measured colorimetrically by the formazan-based MTS cell viability assay (CellTiter 96 AQueous One Solution Cell Proliferation Assay from Promega, Madison, WI), and the antiviral activity was expressed as the 50% effective concentration (EC_50_). In parallel, the 50% cytotoxic concentration (CC_50_) was derived from the mock-infected cells. The activities were compared with the activities of reference antiviral drugs: remdesivir, ribavirin, zanamivir, rimantadine and brivudine (BVDU).

## Figures and Tables

**Table 1:** In vitro antiproliferative activity against a broad panel of cancer cell lines.

Cpd	IC_50_ (μM)							
	Cell Lines							
	LN-229	Capan-1	HCT-116	NCI-H460	DND-41	HL-60	K-562	Z-138
**5**	>100	98.0 ± 2.9	>100	>100	>100	81.7±26	>100	>100
**6**	>100	>100	>100	>100	>100	>100	>100	>100
**8**	>100	>100	>100	>100	>100	>100	>100	>100
**9**	>100	>100	>100	>100	>100	>100	>100	>100
**10**	>100	>100	>100	>100	>100	>100	>100	>100
**11**	81.7 ± 17.4	74.5 ± 36.1	>100	84.9 ± 21.4	48.8 ± 12.5	>100	>100	76.4 ± 33.4
**12**	>100	>100	>100	>100	>100	>100	>100	>100
**13**	>100	>100	>100	>100	>100	>100	>100	>100
**14**	8.0 ± 1.8	9.4 ± 0.4	13.6 ± 2.8	13.0 ± 7.6	10.8 ± 0.4	9.5 ± 1.3	49.7 ± 3.1	8.5 ± 2.0
**15**	73.5 ± 0.8	>100	>100	>100	17.0 ± 1.3	>100	>100	29.1 ± 16.5
**16**	52.4 ± 8.1	52.7 ± 2.9	77.2 ± 19.1	>100	11.9 ± 0.1	91.4 ± 5.1	53.6 ± 2.8	12.1 ± 0.8
**17**	>100	>100	>100	>100	>100	>100	>100	>100
**18**	>100	>100	>100	>100	>100	>100	>100	>100
**19**	>100	>100	>100	>100	>100	>100	>100	>100
**DTX**	0.0041 ± 0.003	0.0038 ± 0.003	0.0025 ± 0.000	0.0034 ± 0.000	0.0025 ± 0.000	0.0022 ± 0.000	0.0085 ± 0.001	0.0023 ± 0.000
**STS**	0.0668 ± 0.016	0.0519 ± 0.021	0.0701 ± 0.006	0.0448 ± 0.004	0.0548 ± 0.001	0.0586 ± 0.018	0.0374 ± 0.006	0.0484 ± 0.007

**Table 2:** In vitro antiproliferative activity against selected cancer cell lines.

Cpd	IC_50_ (μM)		
	Cell Lines		
	PC3	HeLa	SW 620
**5**	>100	13.6 ± 3.4	>100
**6**	>100	28.9 ± 7.2	>100
**8**	3.2 ± 0.7	1.8 ± 0.02	2.0 ± 0.2
**9**	>100	>100	>100
**10**	≥ 100	1.3 ± 0.26	0.4 ± 0.1
**11**	≥ 100	11.1 ± 3.5	12.1 ± 3.7
**12**	>100	>100	>100
**13**	>100	>100	>100
**14**	1.5 ± 0.5	4.3 ± 2.6	0.7 ± 0.3
**15**	8.6 ± 0.7	7.3 ± 0.4	3.5 ± 0.2
**16**	13.3 ± 2.6	13.3 ± 4.3	7.4 ± 1.1
**17**	>100	>100	>100
**18**	>100	48.0 ± 0.2	>100
**19**	28.8 ± 15.0	26.1 ± 0.01	63.8 ± 7.4
**etoposide**	11.6 ± 1.9	0.004 ± 0.001	0.63 ± 0.1
**doxorubicine**	0.13 ± 0.02	0.16 ± 0.05	0.02 ± 0.1

**Table 3:** Antibacterial activity in vitro.

Cpd	MIC (μM)			
	*S. aureus* ATCC 29213	*E. coli* ATCC 25922	*E. coli* Efflux Del	*S. Pneumoniae* ATCC 49619
**5**	>64	>64	>64	>64
**6**	>64	>64	>64	>64
**7**	>64	>64	>64	>64
**8**	>64	>64	>64	>64
**9**	>64	>64	>64	>64
**10**	>64	>64	>64	>64
**11**	>64	>64	>64	>64
**12**	>64	>64	>64	>64
**13**	>64	>64	>64	>64
**14**	>64	>64	32	>64
**15**	>64	>64	>64	>64
**16**	>64	>64	>64	>64
**17**	>64	>64	>64	>64
**18**	>64	>64	>64	>64
**19**	>64	>64	>64	>64
**Ampicillin**	1	1	2	<0.125
**Ceftazidime**	16	0.25	0.25	0.25
**Ciprofloxacin**	0.25	<0.125	<0.125	0.25
**Meropenem**	<0.125	<0.125	<0.125	<0.125

**Table 4:** Cytotoxicity in the virus host cell lines.

Cpd	CC_50_ µM		
	HEL 299	Huh7	MDCK
**5**	72.8 ± 38.5	>100	79.2 ± 29.4
**6**	>100	>100	>100
**7**	>100	>100	>100
**8**	>100	>100	>100
**9**	>100	>100	>100
**10**	>100	>100	>100
**11**	>100	34.9 ± 6.7	>100
**12**	>100	>100	>100
**13**	81.1 ± 26.8	>100	>100
**14**	9.3 ± 2.3	<0.8	33.9 ± 0.1
**15**	>100	50.3	>100
**16**	>100	3.0 ± 2.1	71.2 ± 40.8
**17**	>100	>100	>100
**18**	>100	>100	>100
**19**	>100	>100	>100
**Remdesivir**	>10	>10	-
**Ribavirin**	>250	10.7 ± 2.6	80.2 ± 18.6
**Zanamivir**	-	-	>100
**Rimantadine**	-	-	>100
**BVDU**	-	-	>100

**Table 5:** In vitro antiviral activity.

Cpd	EC_50_ (μM)										
	Viruses										
	HCoV 229E HEL 299	HCoV OC43 HEL 299	HCoV NL63 Huh7	Influenza H1N1 MDCK	Influenza H3N2 MDCK	Influenza B MDCK	RSV A Long HEL 299	HSV-1 KOS HSV-1	YFV 17D Huh7	Zika mr766 Huh7	Sindbis Huh7
**7**	>100	>100	>100	>100	>100	>100	21.0 ± 5.9	>100	>100	>100	>100
**8**	>100	>100	>100	72.35 ± 8.0	35.85 ± 12.4	56.7 ± 10.2	>100	>100	>100	>100	>100
**10**	>100	>100	>100	71.35 ± 24.3	79.2 ± 14.7	93.95 ± 5.2	86.9 ± 18.6	>100	>100	>100	>100
**17**	>100	>100	>100	>100	>100	>100	79.0 ± 29.7	>100	>100	>100	>100
**Remdesivir**	0.06	0.06	0.03	-	-	-	0.03 ± 0.01	-	6.2	0.7	>10
**Ribavirin**	82.6	170.1	>250	10.5 ± 3.8	4 ± 2.7	2.8 ± 1.8	10.8 ± 2.1	-	>250	>250	148.1
**Zanamivir**	-	-	-	0.13 ± 0.04	16.8 ± 6.8	0.05 ± 0.03	-	-	-	-	-
**Rimantadine**	-	-	-	4.4 ± 4.2	0.05 ± 0.05	>100	-	-	-	-	-
**BVDU**	-	-	-	-	-	-	-	0.05	-	-	-

## Supplementary Materials

The following are available online at https://www.mdpi.com/article/10.3390/molecules28010034/s1, Figures S1–S30: NMR spectra of prepared compounds.
