# Supplementary material for: Biological Activity of Amidino-Substituted Imidazo [4,5-b]pyridines
Source: Molecules. 2022 Dec 21;28(1):34. doi: 10.3390/molecules28010034 (PMC9822021; doi:10.3390/molecules28010034)
Supplement: Supplementary file 1 [file molecules-28-00034-s001.zip › molecules-2064925-supplementary.pdf]

Biological Activity of Amidino Substituted Imidazo [4,5-*b*]pyridines

Ida Boček Pavlinac[1], Katarina Zlatić[2], Leentje Persoons[3], Dirk Daelemans[3], Mihajlo Banjanac[4], Vedrana Radovanović[4], Kristina Butković[5], Marijeta Kralj[2], and Marijana Hranjec[1*]

[1]Faculty of Chemical Engineering and Technology, University of Zagreb, 10000 Zagreb, Croatia; [2]Division of Molecular Medicine, Institute Ruđer Bošković, 10000 Zagreb, Croatia;

[3]KU Leuven Department of Microbiology, Immunology and Transplantation, Laboratory of Virology and Chemotherapy, Rega Institute, 3000 Leuven, Belgium;

[4]Pharmacology *in vitro*, Selvita Ltd., 10000 Zagreb, Croatia;

[5]Chemistry, Selvita Ltd., 10000 Zagreb, Croatia;

Content

Figures S1-S30: NMR spectra of prepared compounds

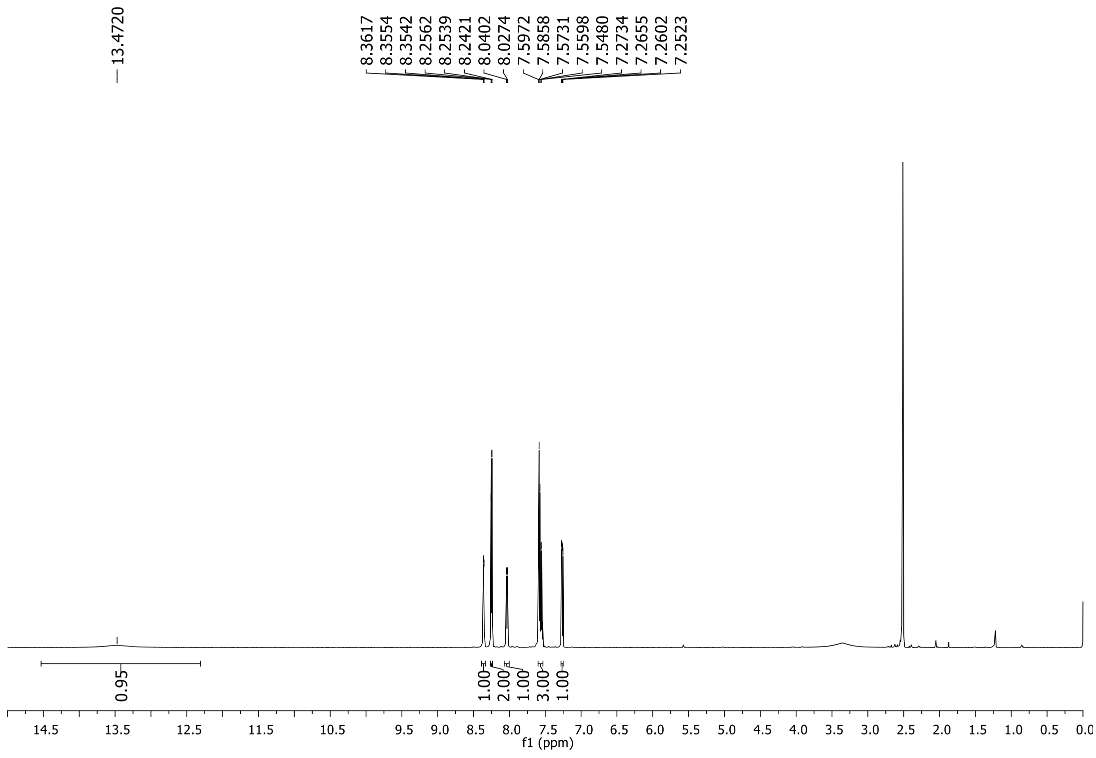

Figure S1. ^{1}H NMR spectrum (DMSO-d_6, 300 MHz) of 2-phenyl-1*H*-imidazo[4,5-*b*]pyridine **5**

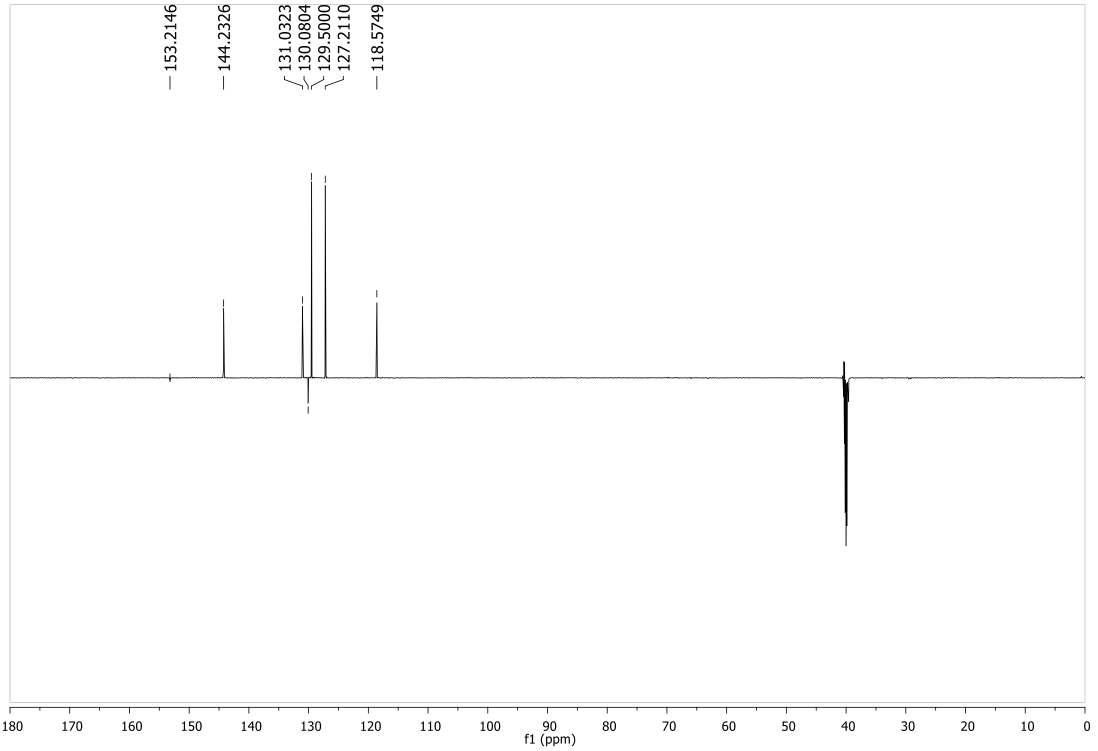

Figure S2. ^{13}C NMR spectrum (DMSO-d_6, 75 MHz) of 2-phenyl-1*H*-imidazo[4,5-*b*]pyridine **5**

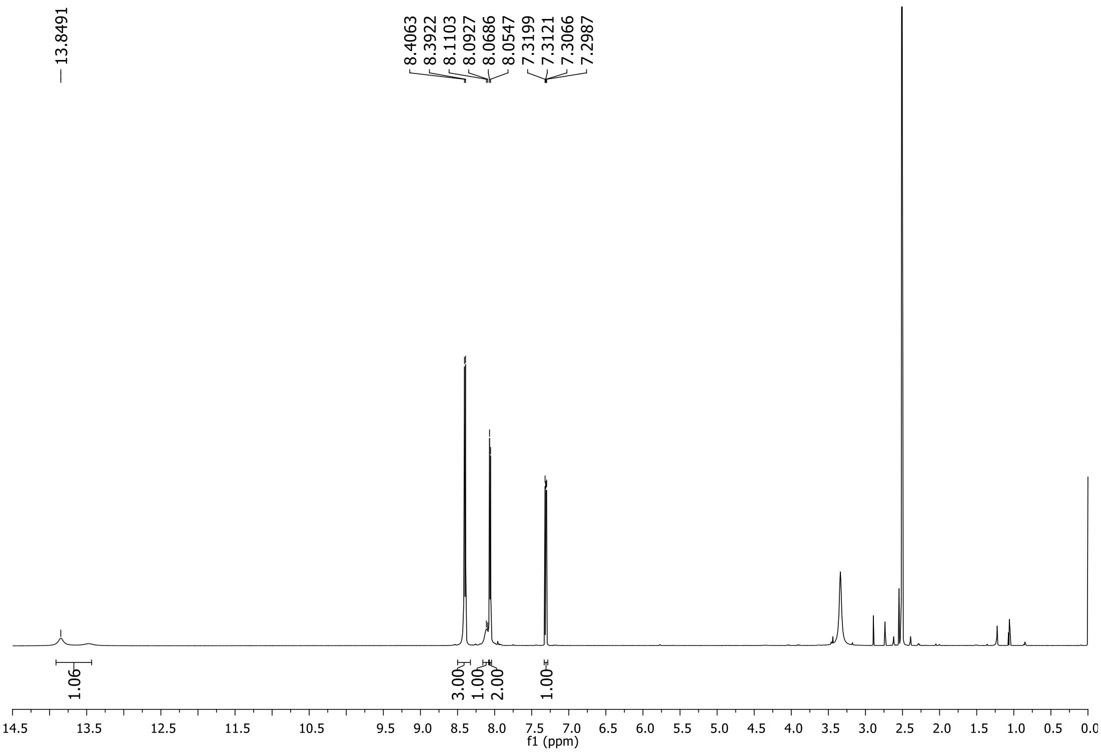

Figure S3. ^{1}H NMR spectrum (DMSO-d_6, 600 MHz) of 4-(1*H*-imidazo[4,5-*b*]pyridin-2-yl)benzonitrile **6**

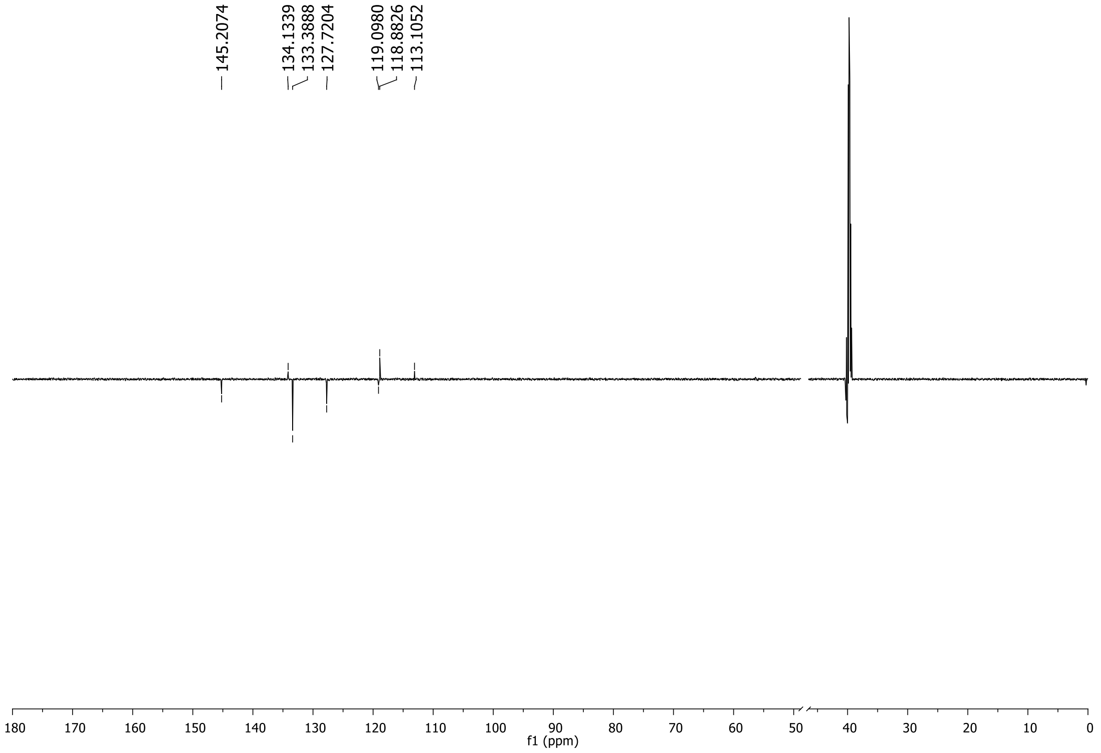

Figure S4. ^{13}C NMR spectrum (DMSO-d_6, 151 MHz) of 4-(1*H*-imidazo[4,5-*b*]pyridin-2-yl)benzonitrile **6**

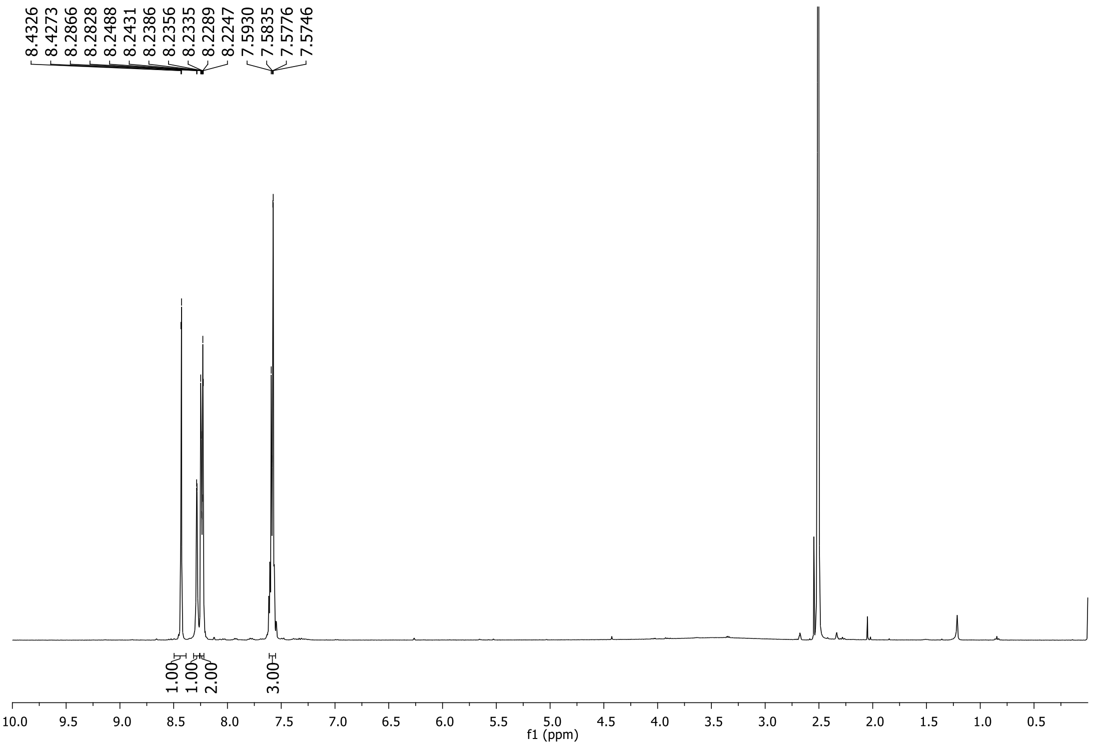

Figure S5. ^{1}H NMR spectrum (DMSO-d_6, 400 MHz) of 6-bromo-2-phenyl-1*H*-imidazo[4,5-*b*]pyridine **7**

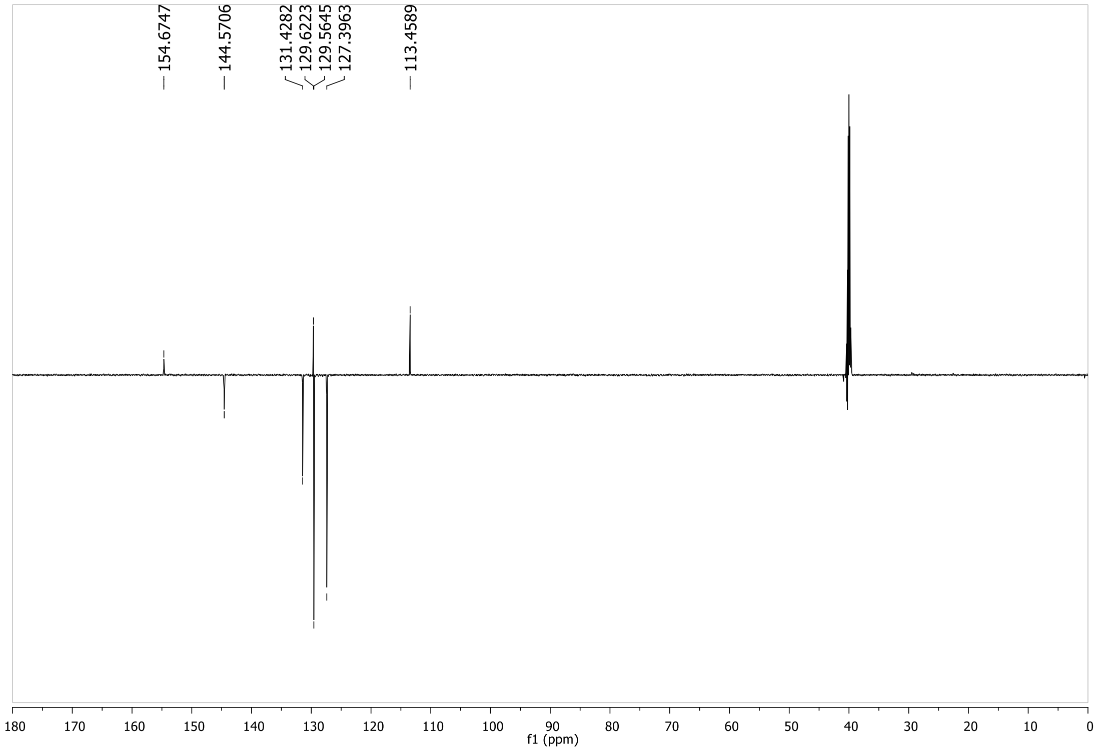

Figure S6. ^{13}C NMR spectrum (DMSO-d_6, 151 MHz) of 6-bromo-2-phenyl-1*H*-imidazo[4,5-*b*]pyridine **7**

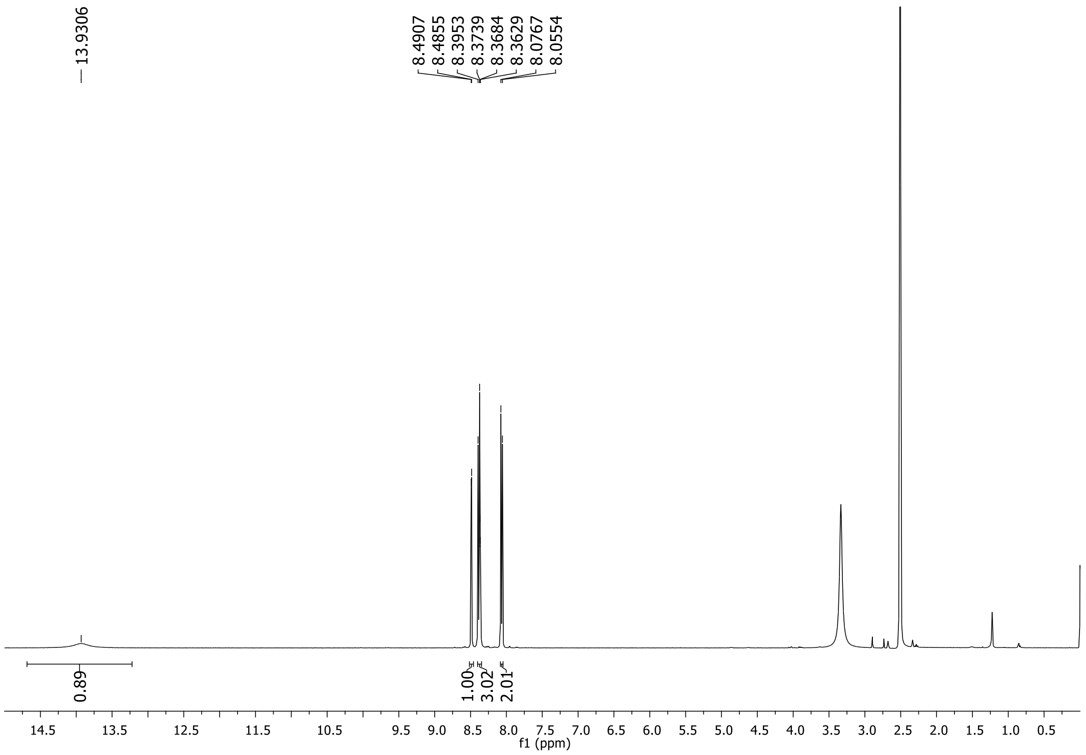

Figure S7. ^{1}H NMR spectrum (DMSO-d_6, 600 MHz) of 4-(6-bromo-1*H*-imidazo[4,5-*b*]pyridin-2-yl)benzonitrile **8**

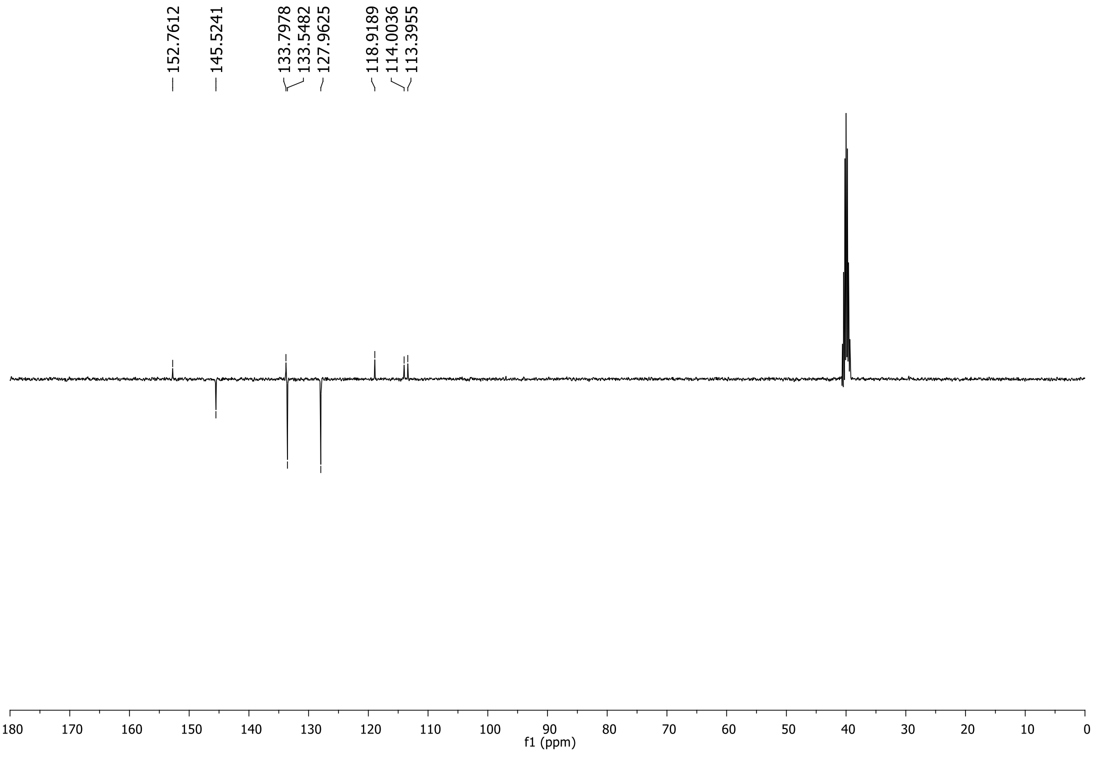

Figure S8. ^{13}C NMR spectrum (DMSO-d_6, 101 MHz) of 4-(6-bromo-1*H*-imidazo[4,5-*b*]pyridin-2-yl)benzonitrile **8**

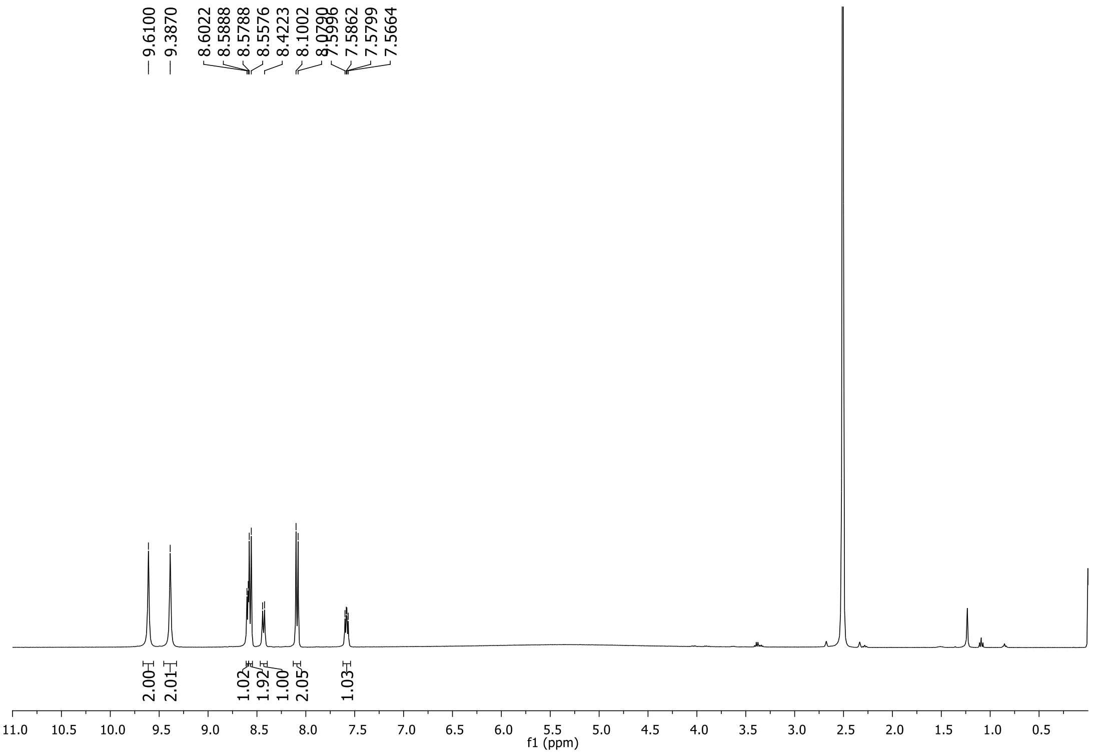

Figure S9. ^{1}H NMR spectrum (DMSO-d_6, 400 MHz) of 4-(1*H*-imidazo[4,5-*b*]pyridin-2-yl)benzimidamide hydrochloride **9**

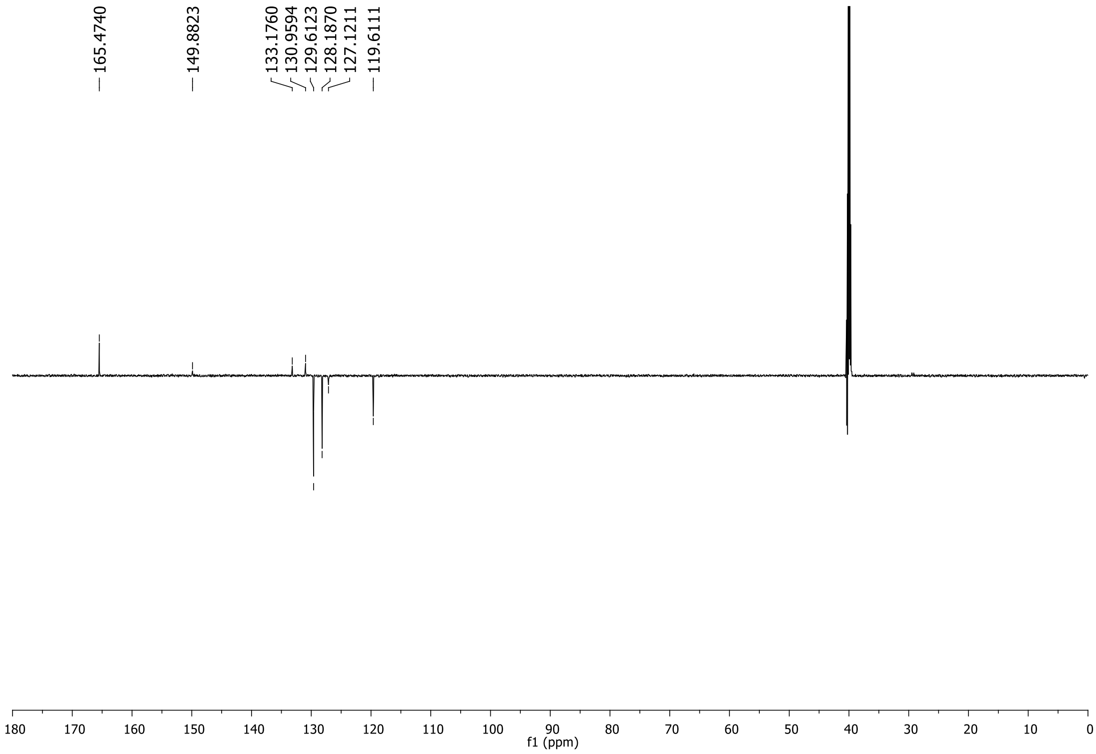

Figure S10. ^{13}C NMR spectrum (DMSO-d_6, 151 MHz) of 4-(1*H*-imidazo[4,5-*b*]pyridin-2-yl)benzimidamide hydrochloride **9**

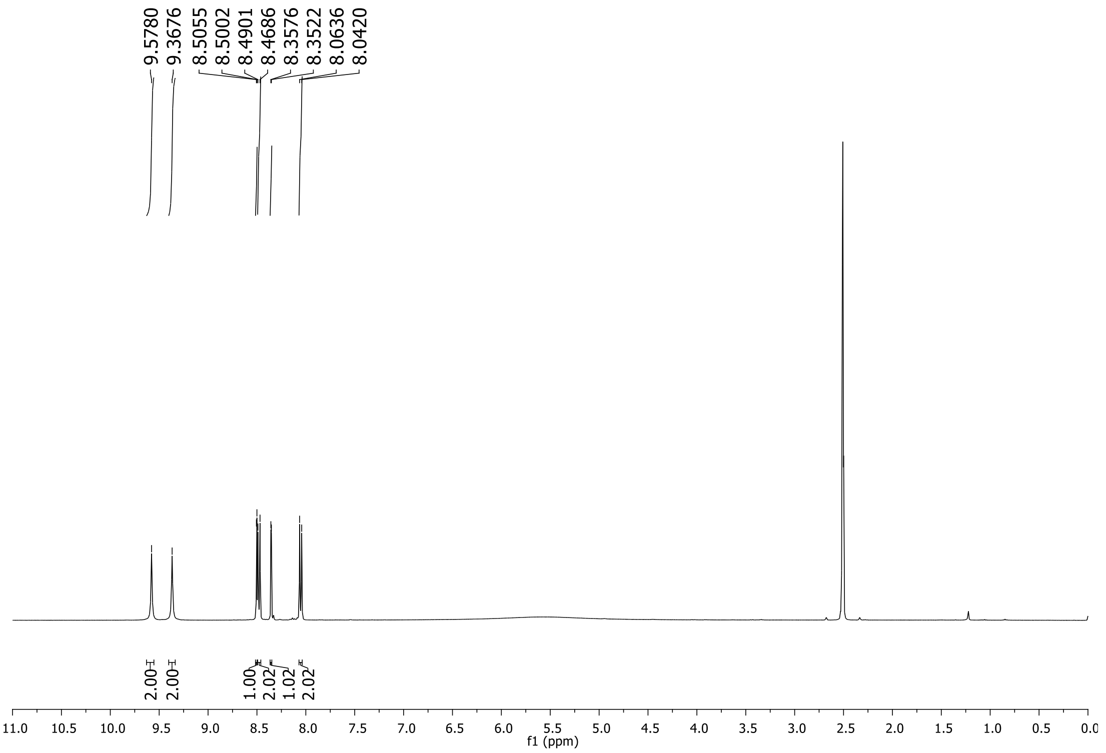

Figure S11. ^{1}H NMR spectrum (DMSO-d_6, 400 MHz) of 4-(6-bromo-1*H*-imidazo[4,5-*b*]pyridin-2-yl)benzimidamide hydrochloride **10**

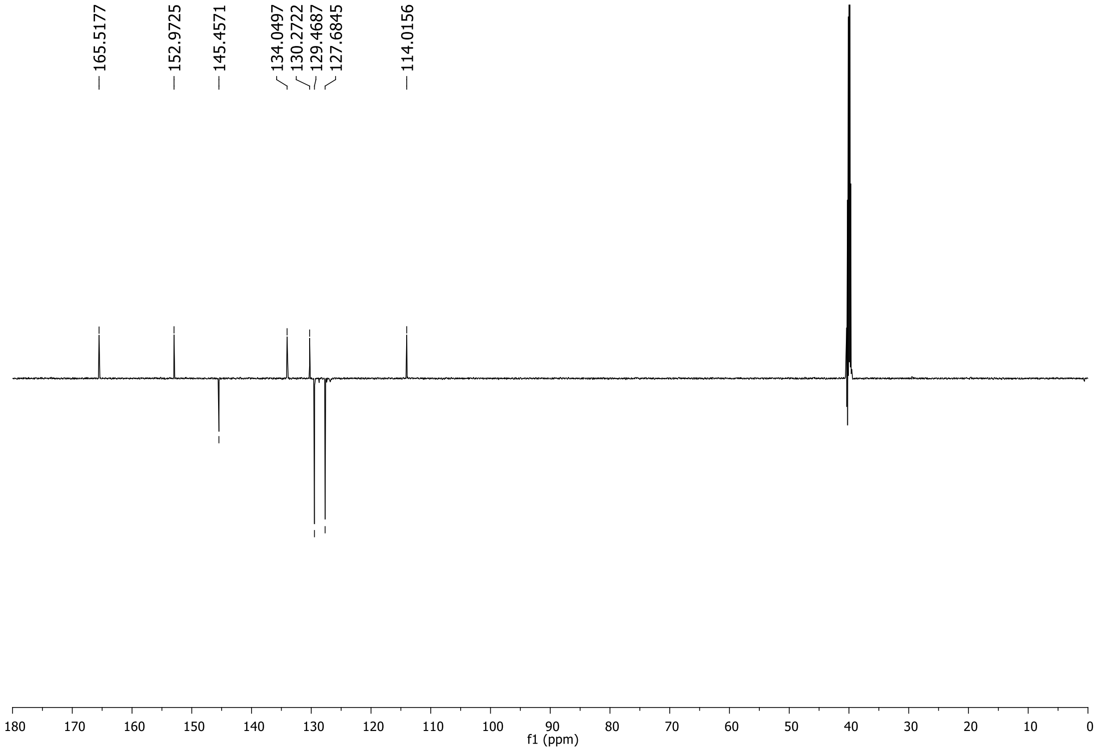

Figure S12. ^{13}C NMR spectrum (DMSO-d_6, 151 MHz) of 4-(6-bromo-1*H*-imidazo[4,5-*b*]pyridin-2-yl)benzimidamide hydrochloride **10**

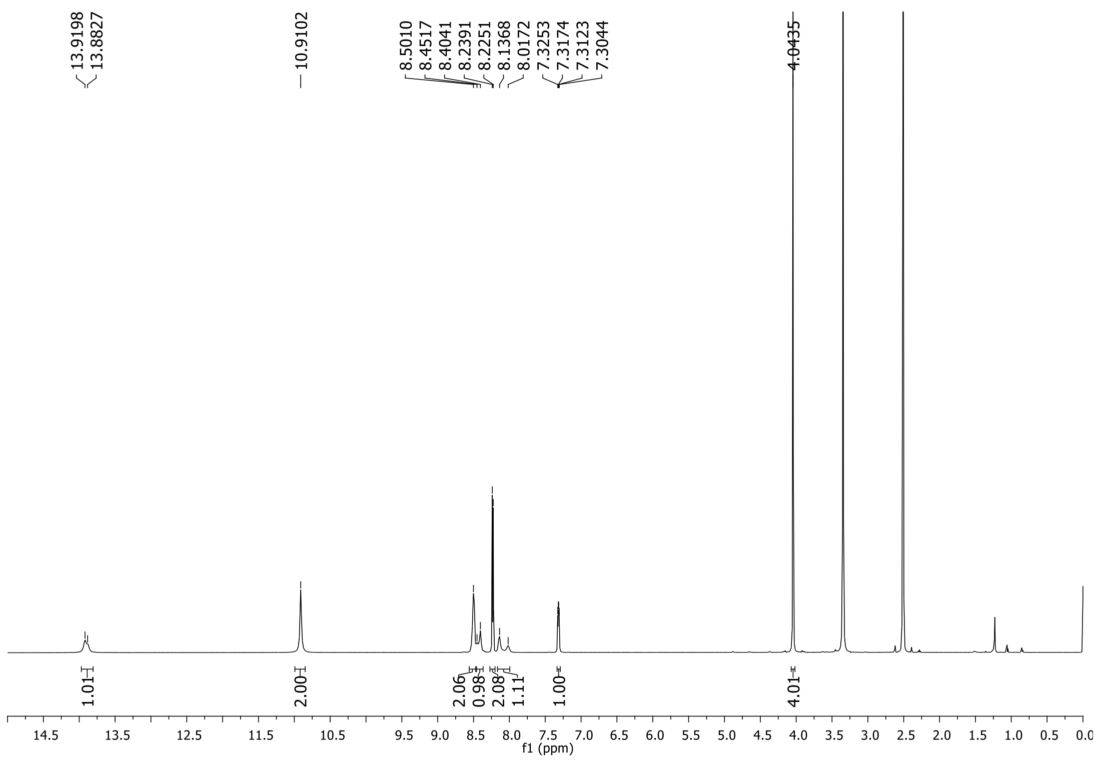

Figure S13. ^{1}H NMR spectrum (DMSO-d_6, 600 MHz) of 2-(4-(4,5-dihydro-1*H*-imidazol-2-yl)phenyl)-1H-imidazo[4,5-*b*]pyridine hydrochloride **11**

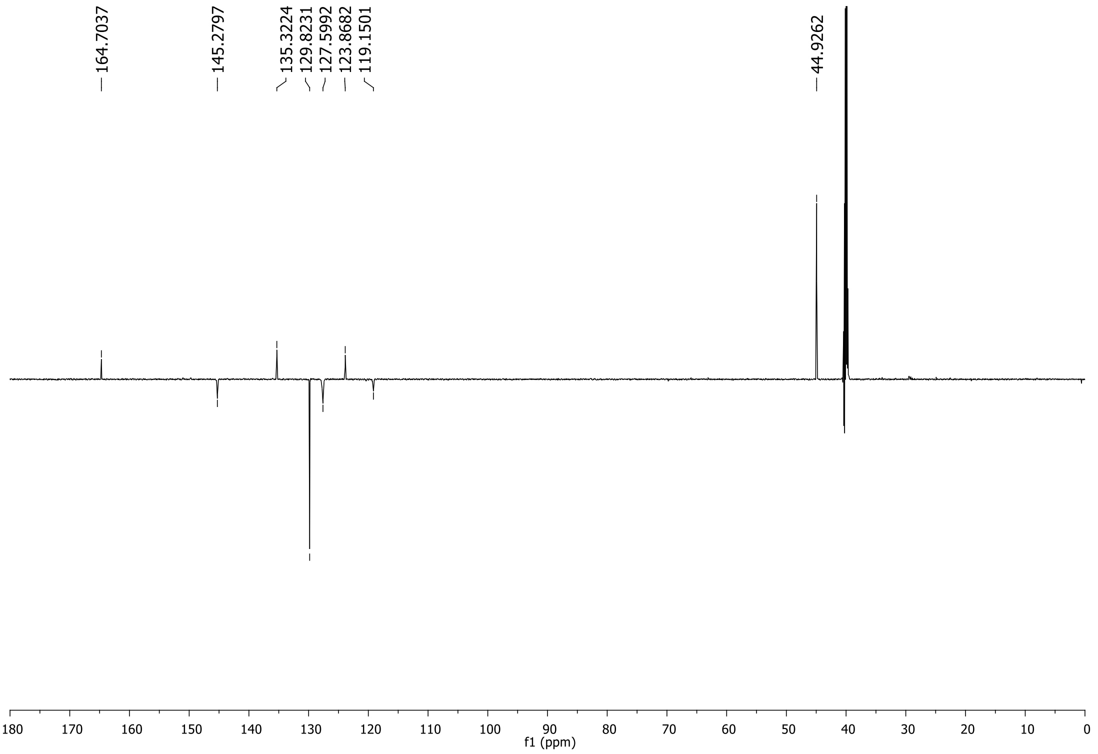

Figure S14. ^{13}C NMR spectrum (DMSO-d_6, 151 MHz) of 2-(4-(4,5-dihydro-1*H*-imidazol-2-yl)phenyl)-1H-imidazo[4,5-*b*]pyridine hydrochloride **11**

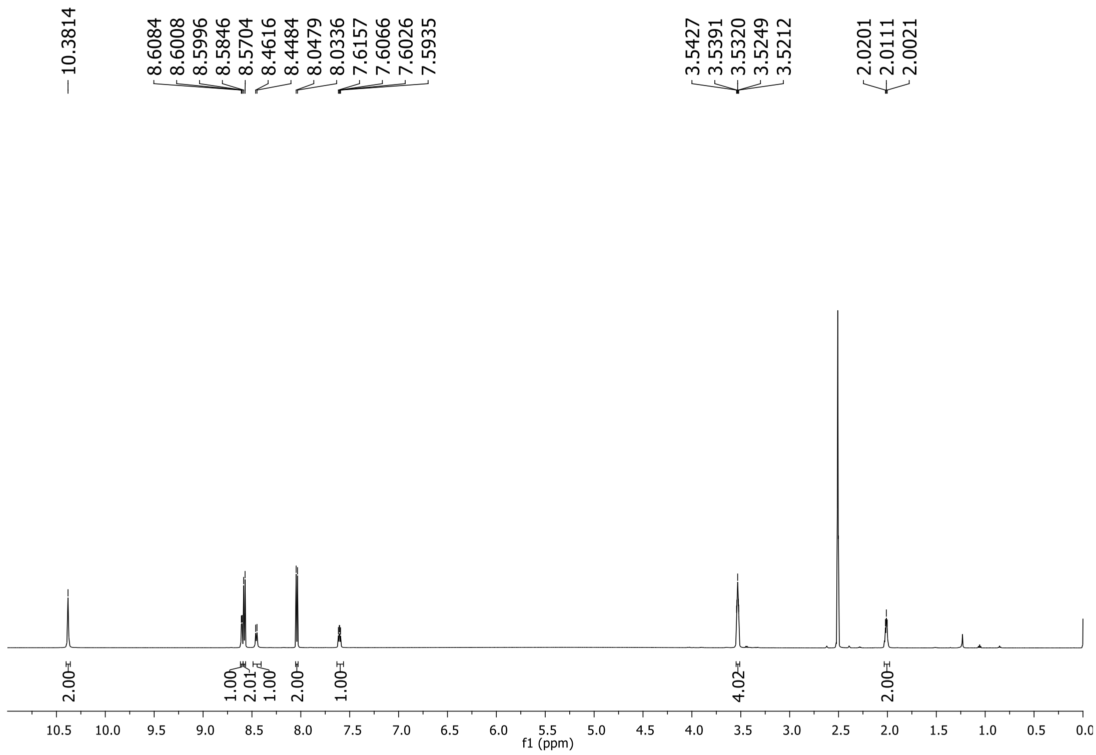

Figure S15. ^{1}H NMR spectrum (DMSO-d_6, 600 MHz) of 2-(4-(1*H*-imidazo[4,5-*b*]pyridin-2-yl)phenyl)-3,4,5,6-tetrahydropyrimidin-1-ium chloride **12**

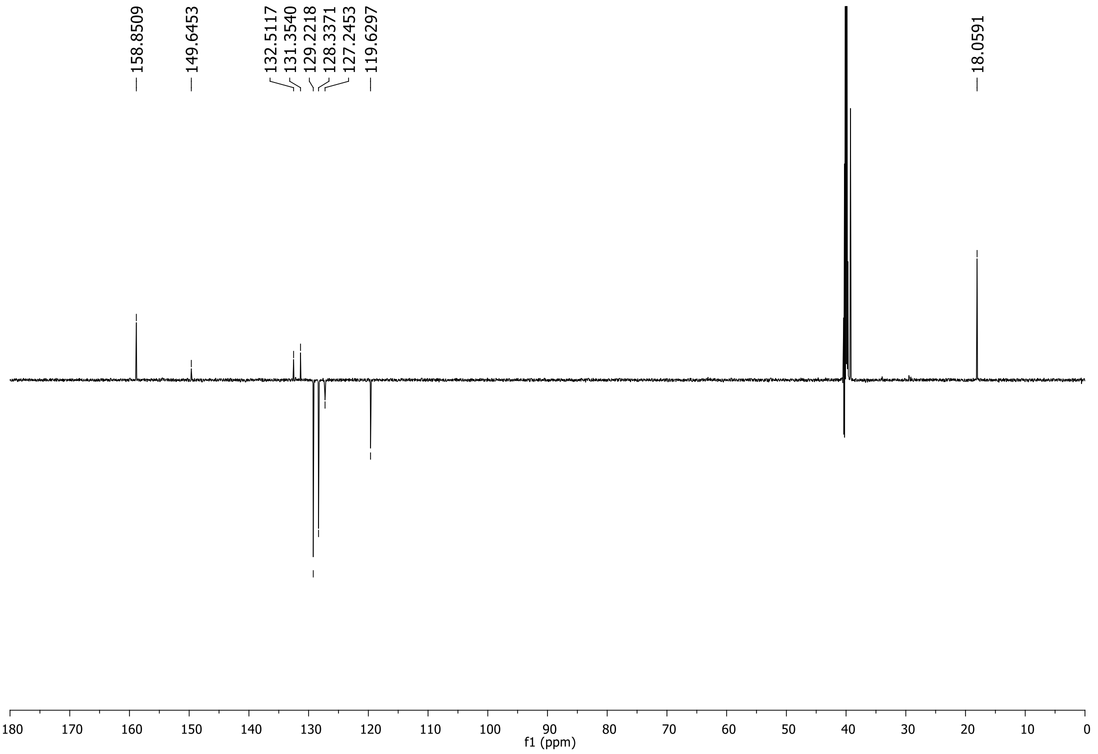

Figure S16. ^{13}C NMR spectrum (DMSO-d_6, 151 MHz) of 2-(4-(1*H*-imidazo[4,5-*b*]pyridin-2-yl)phenyl)-3,4,5,6-tetrahydropyrimidin-1-ium chloride **12**

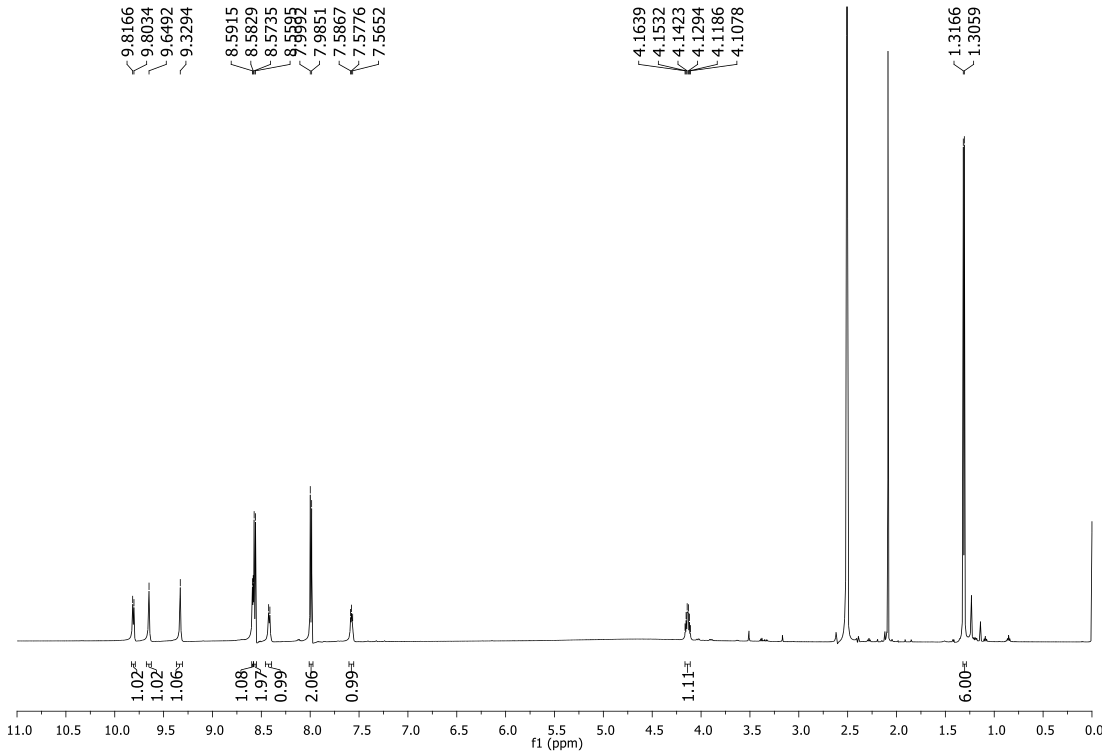

Figure S17. ^{1}H NMR spectrum (DMSO-d_6, 600 MHz) of 4-(1*H*-imidazo[4,5-*b*]pyridin-2-yl)-*N*-isopropylbenzimidamide hydrochloride **13**

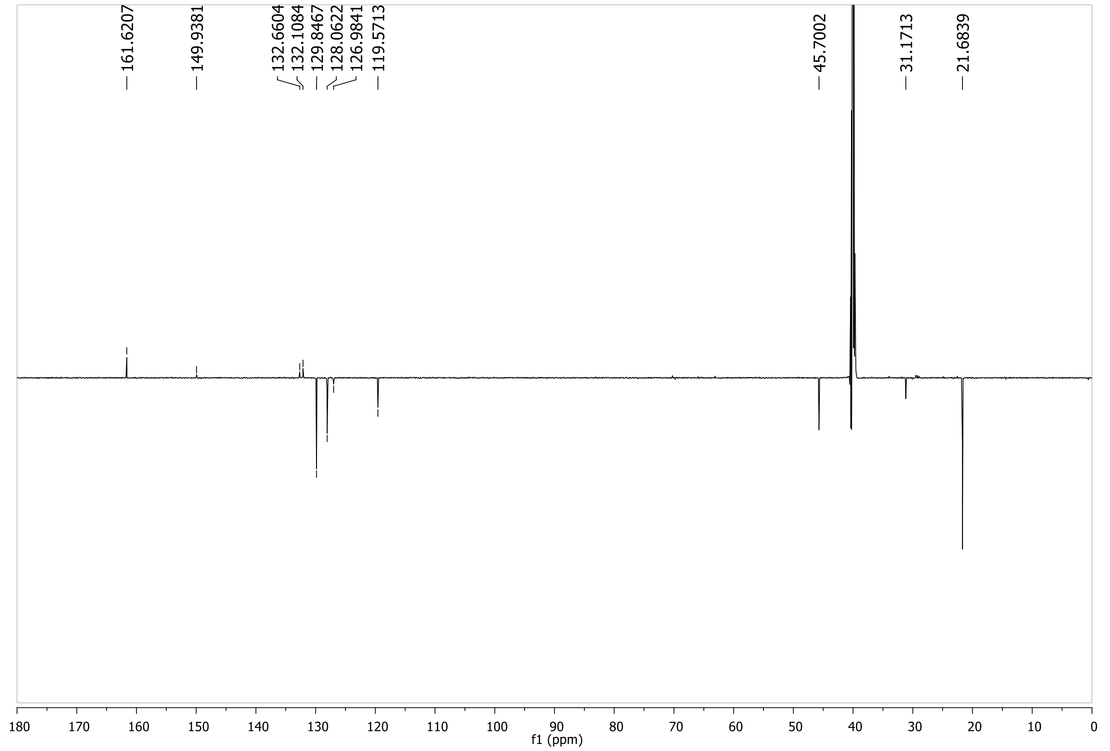

Figure S18. ^{13}C NMR spectrum (DMSO-d_6, 151 MHz) of 4-(1*H*-imidazo[4,5-*b*]pyridin-2-yl)-*N*-isopropylbenzimidamide hydrochloride **13**

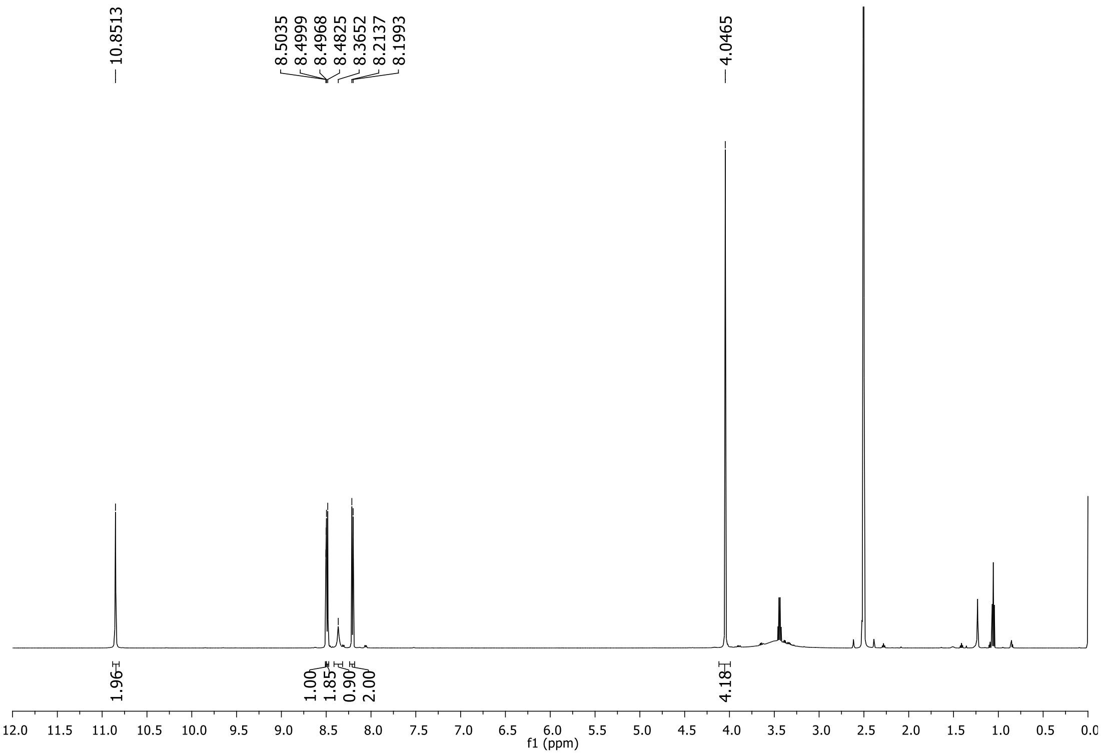

Figure S19. ^{1}H NMR spectrum (DMSO-d_6, 600 MHz) of 6-bromo-2-(4-(4,5-dihydro-1*H*-imidazol-2-yl)phenyl)-1*H*-imidazo[4,5-*b*]pyridine hydrochloride **14**

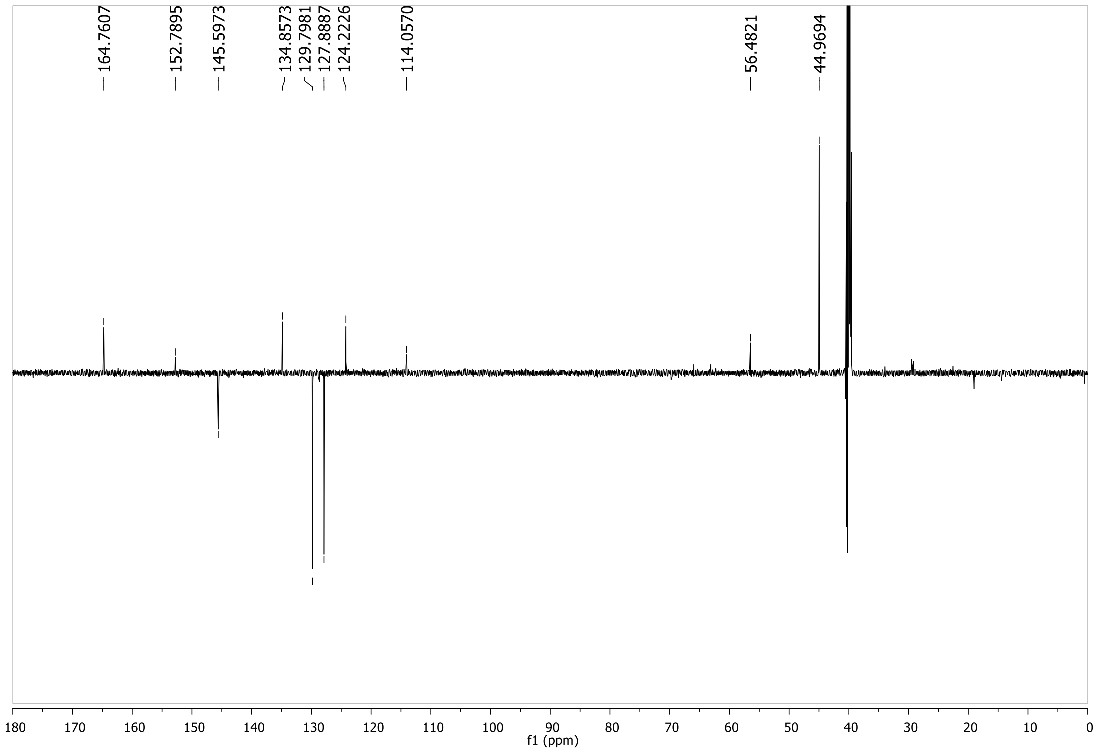

Figure S20. ^{13}C NMR spectrum (DMSO-d_6, 151 MHz) of 6-bromo-2-(4-(4,5-dihydro-1*H*-imidazol-2-yl)phenyl)-1*H*-imidazo[4,5-*b*]pyridine hydrochloride **14**

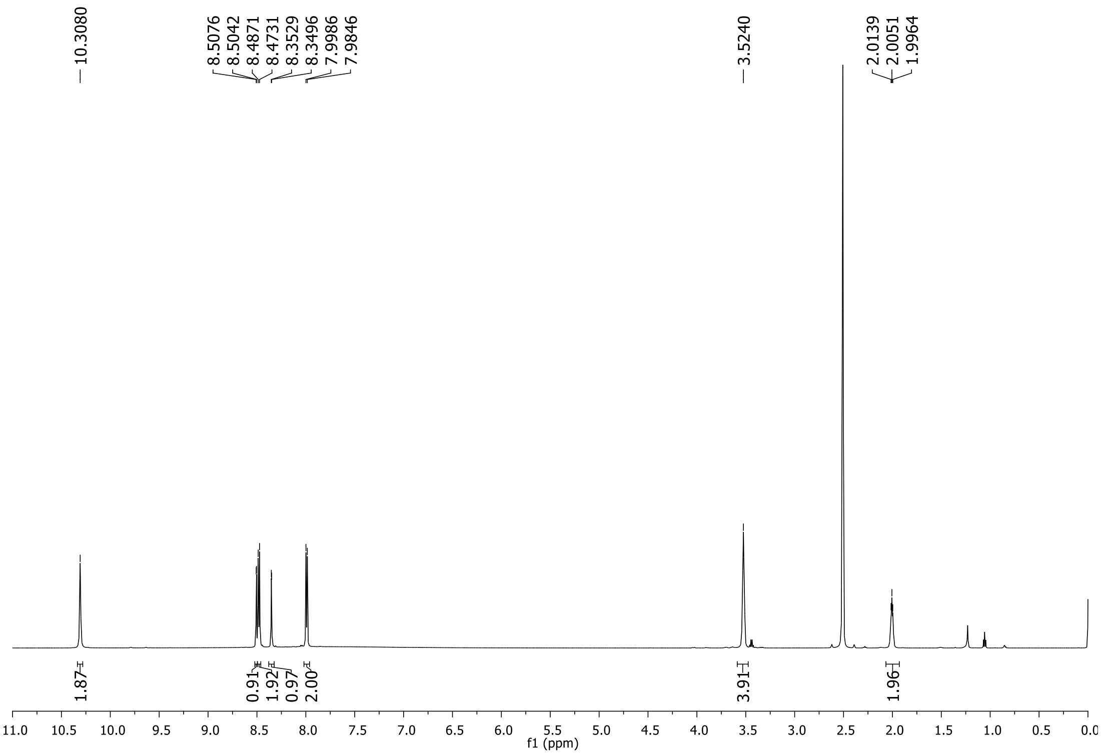

Figure S21. ^{1}H NMR spectrum (DMSO-d_6, 600 MHz) of 6-bromo-2-(4-(1,4,5,6-tetrahydropyrimidin-2-yl)phenyl)-1*H*-imidazo[4,5-*b*]pyridine hydrochloride **15**

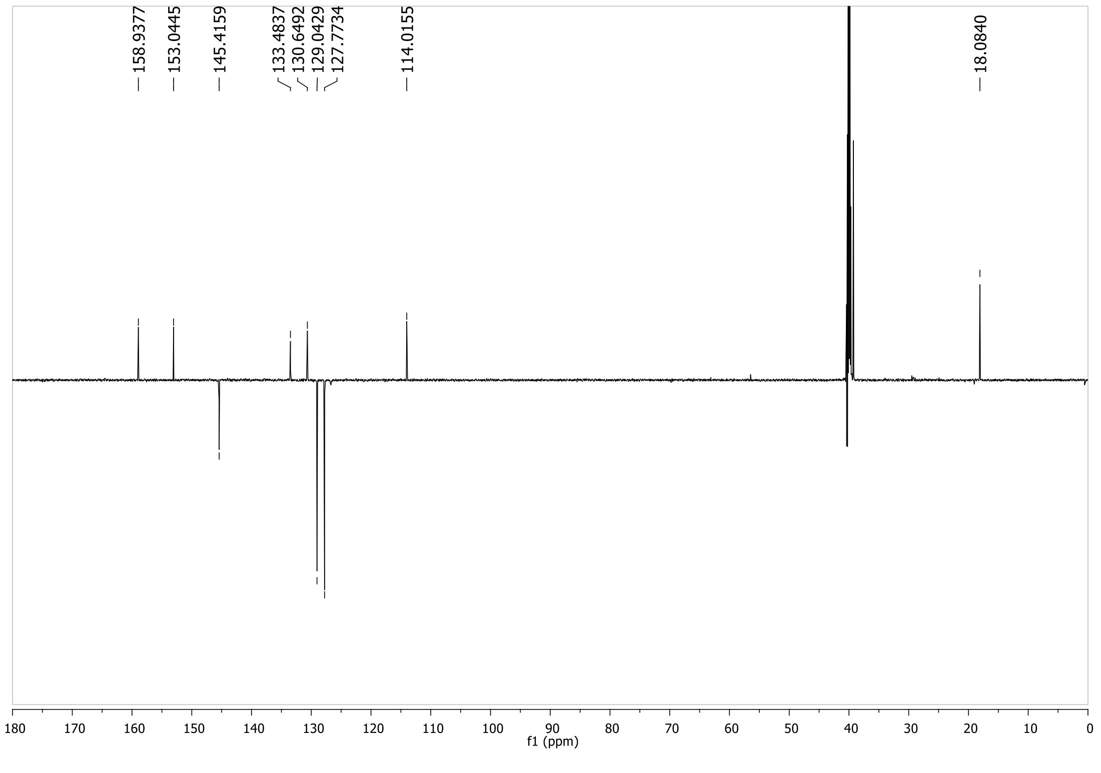

Figure S22. ^{13}C NMR spectrum (DMSO-d_6, 151 MHz) of 6-bromo-2-(4-(1,4,5,6-tetrahydropyrimidin-2-yl)phenyl)-1*H*-imidazo[4,5-*b*]pyridine hydrochloride **15**

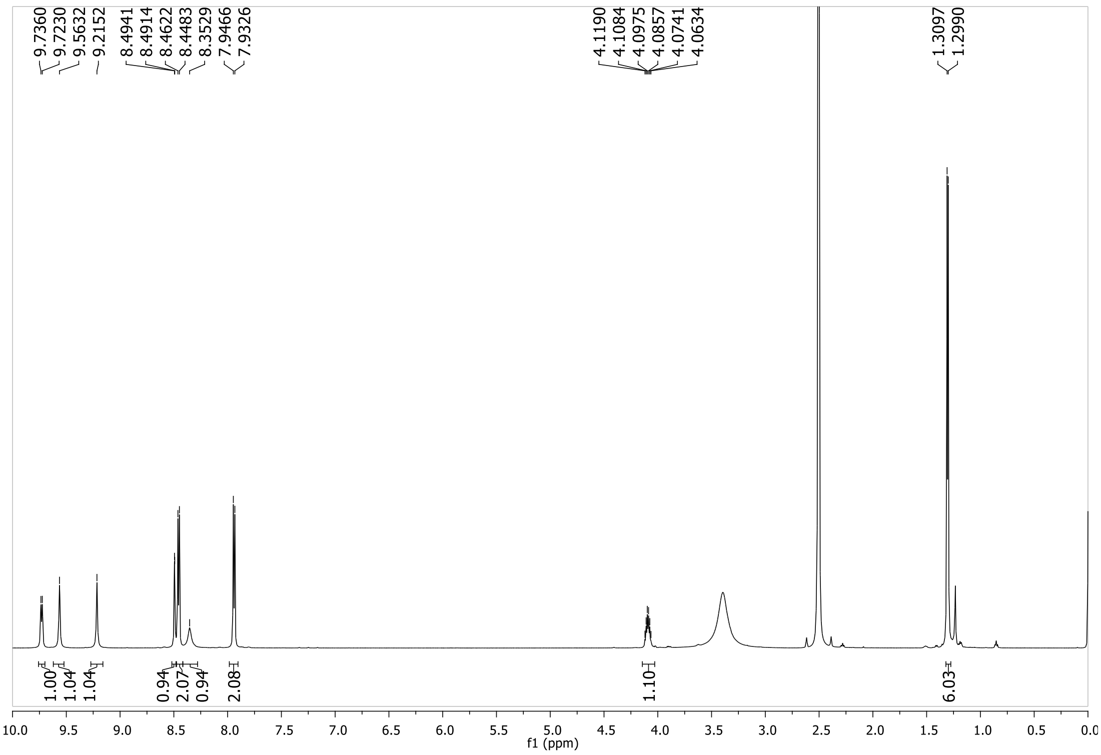

Figure S23. ^{1}H NMR spectrum (DMSO-d_6, 600 MHz) of 4-(6-bromo-1*H*-imidazo[4,5-*b*]pyridin-2-yl)-*N*-isopropylbenzimidamide hydrochloride **16**

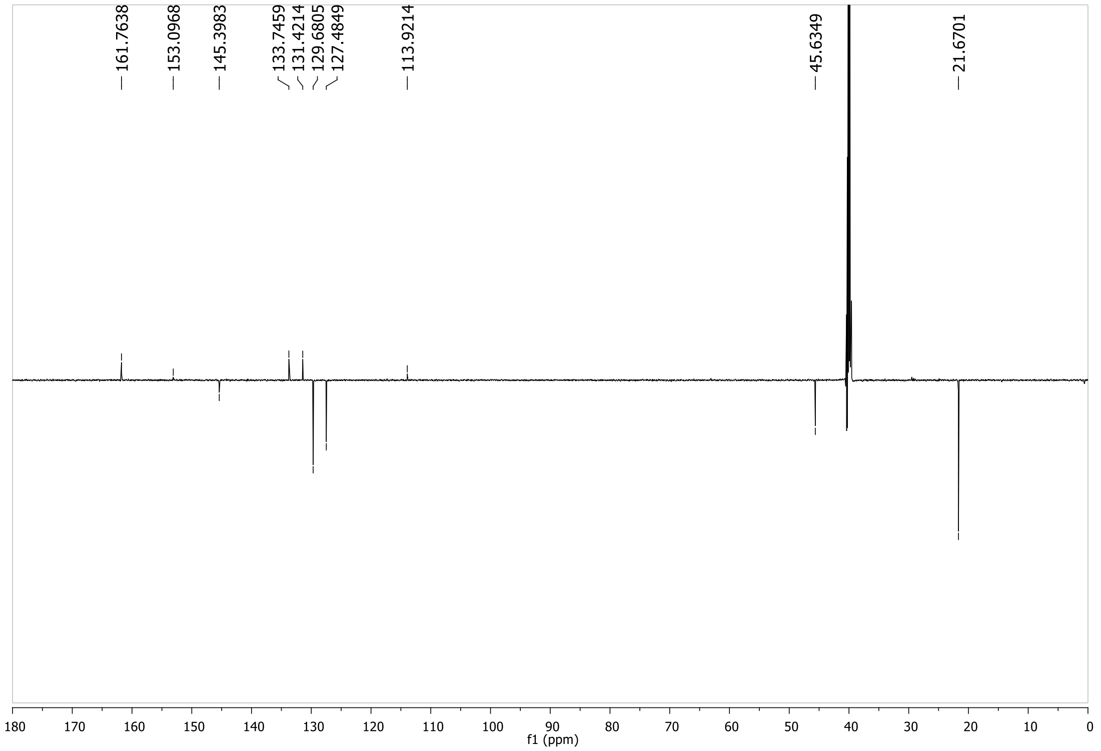

Figure S24. ^{13}C NMR spectrum (DMSO-d_6, 151 MHz) of 4-(6-bromo-1*H*-imidazo[4,5-*b*]pyridin-2-yl)-*N*-isopropylbenzimidamide hydrochloride **16**

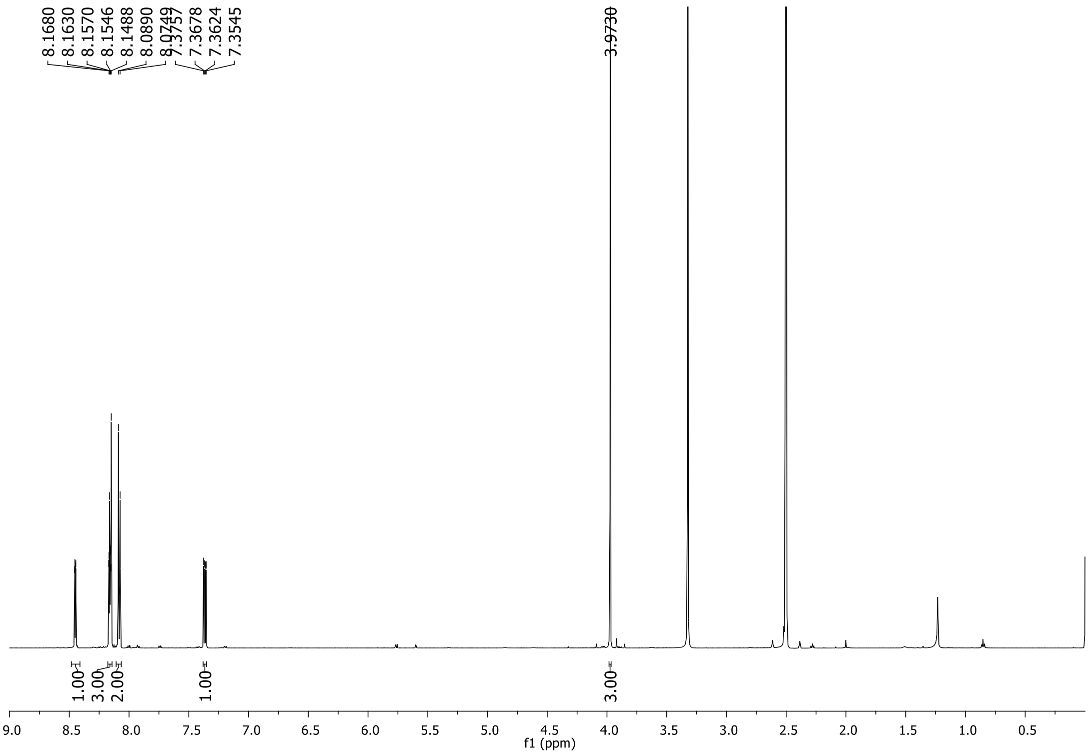

Figure S25. ^{1}H NMR spectrum (DMSO-d_6, 600 MHz) of 4-(3-methyl-3*H*-imidazo[4,5-*b*]pyridin-2-yl)benzonitrile **17**

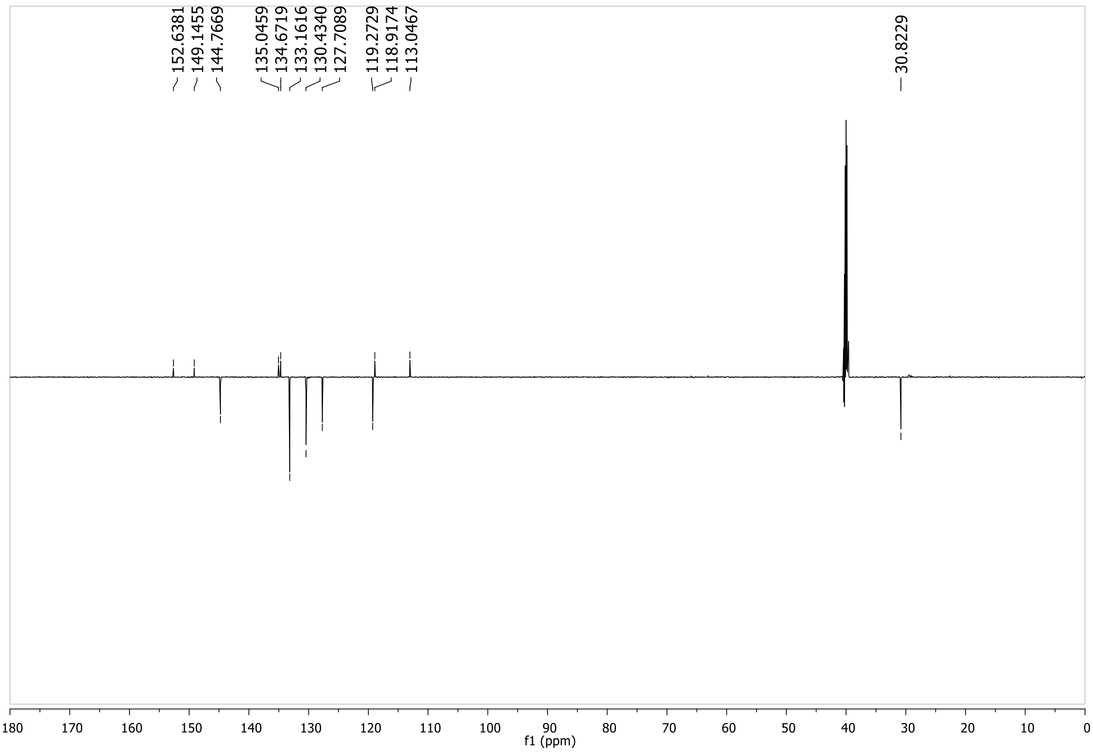

Figure S26. ^{13}C NMR spectrum (DMSO-d_6, 151 MHz) of 4-(3-methyl-3*H*-imidazo[4,5-*b*]pyridin-2-yl)benzonitrile **17**

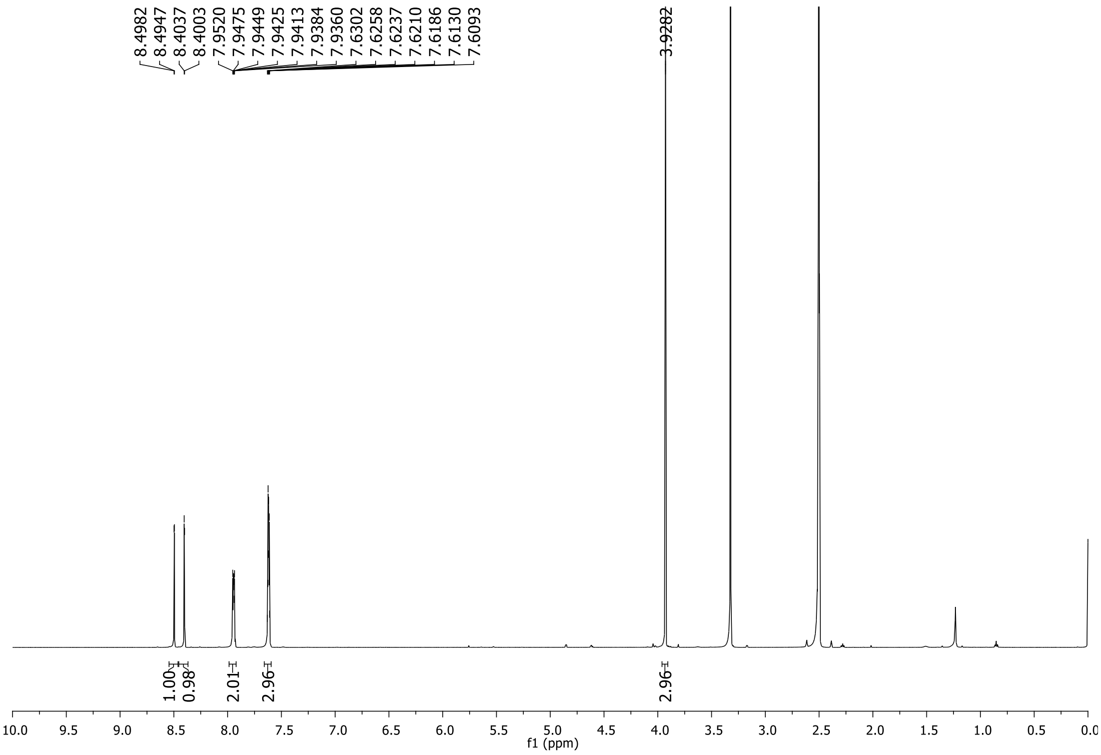

Figure S27. ^{1}H NMR spectrum (DMSO-d_6, 600 MHz) of 6-bromo-3-methyl-2-phenyl-3*H*-imidazo[4,5-*b*]pyridine **18**

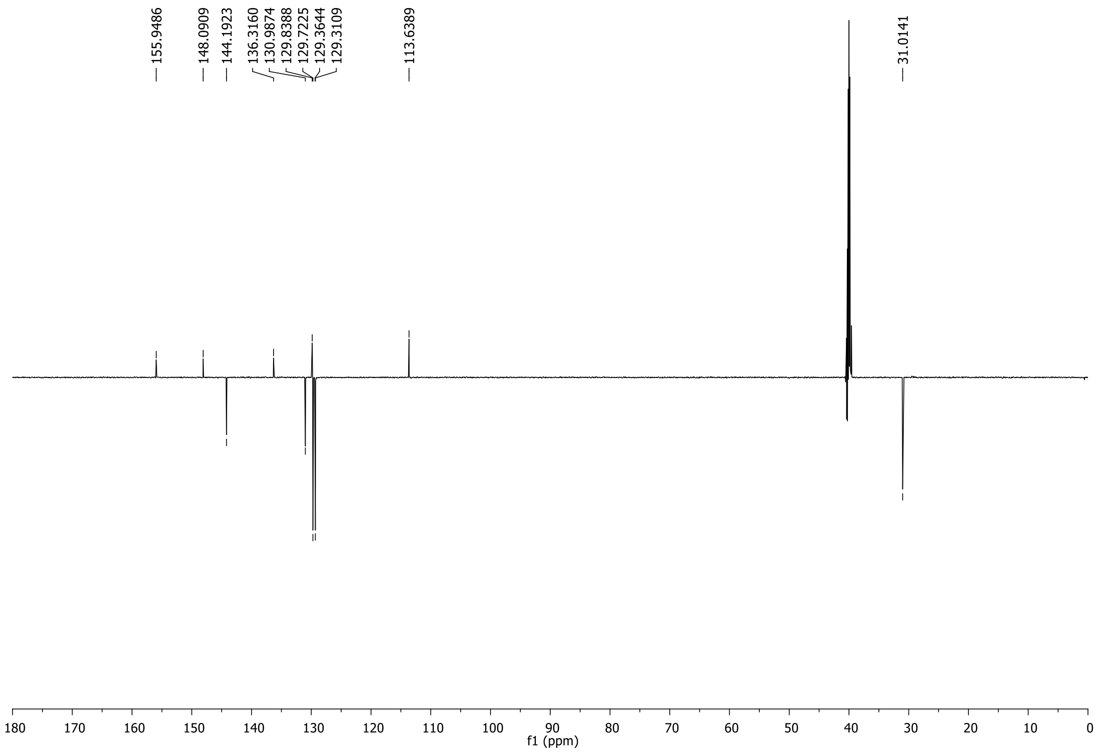

Figure S28. ^{13}C NMR spectrum (DMSO-d_6, 75 MHz) of 6-bromo-3-methyl-2-phenyl-3*H*-imidazo[4,5-*b*]pyridine **18**

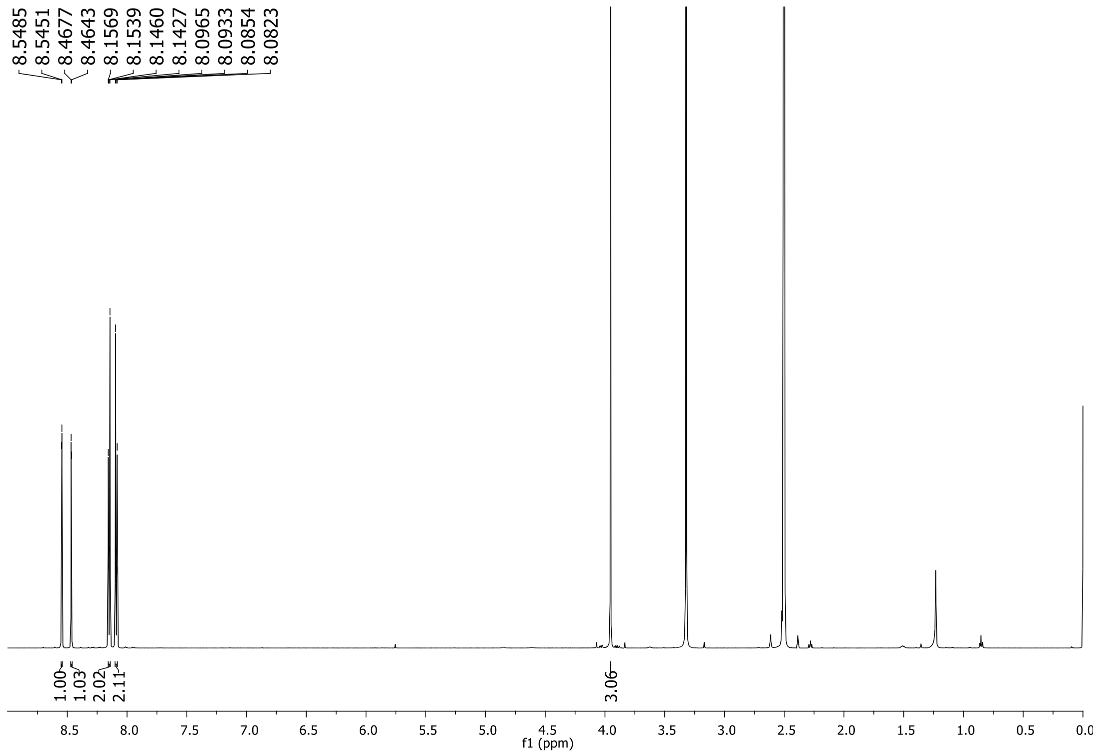

Figure S29. ^{1}H NMR spectrum (DMSO-d_6, 600 MHz) of 4-(6-bromo-3-methyl-3*H*-imidazo[4,5-*b*]pyridin-2-yl)benzonitrile **19**

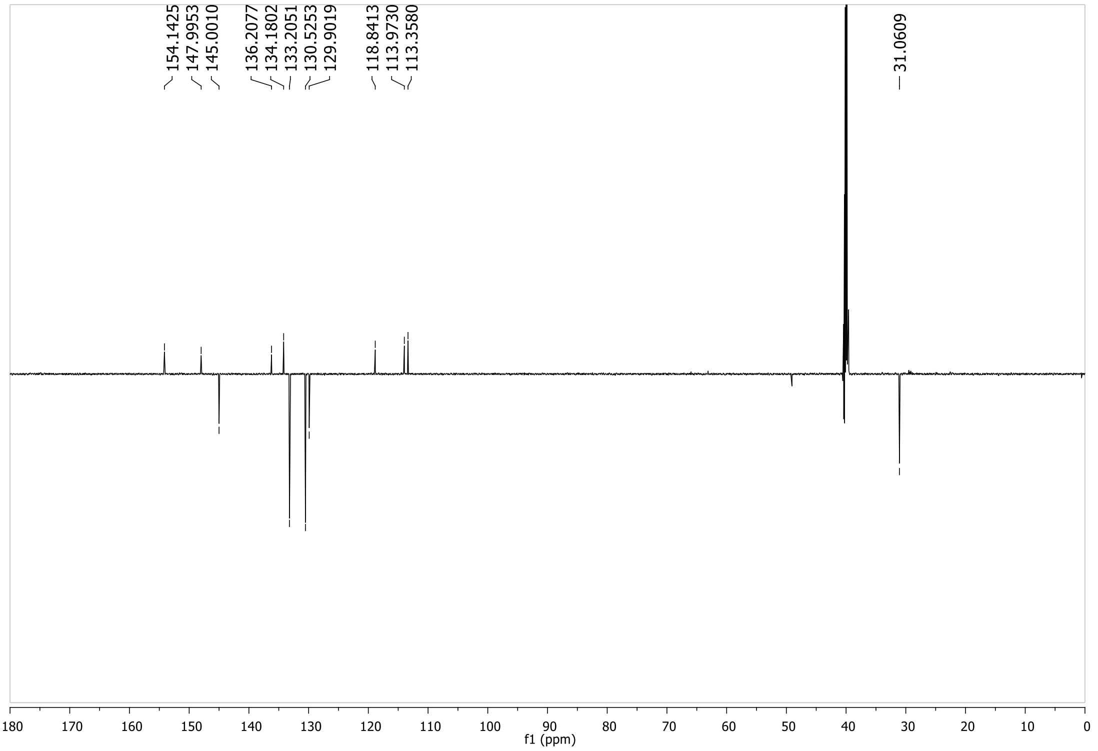

Figure S30. ^{13}C NMR spectrum (DMSO-d_6, 151 MHz) of 4-(6-bromo-3-methyl-3*H*-imidazo[4,5-*b*]pyridin-2-yl)benzonitrile **19**
